# Beyond the laboratory: the bank vole (*Clethrionomys glareolus*) as a novel model organism in biological research

**DOI:** 10.1186/s12983-025-00578-y

**Published:** 2025-09-30

**Authors:** Joanna Górska, Petr Kotlík, Heikki Henttonen, Anna Bajer, Jerzy M. Behnke, Jacek Radwan, Paweł Koteja, Tapio Mappes, Maciej Grzybek

**Affiliations:** 1 Department of Tropical Parasitology, Institute of Maritime and Tropical Medicine, Medical University of Gdansk, Powstania Styczniowego 9B, 81-519 Gdynia, Poland; 2 Institute of Animal Physiology and Genetics, Czech Academy of Sciences, Liběchov, Czech Republic; 3 Natural Resources Institute Finland, Helsinki, Finland; 4 Warsaw University, Warsaw, Poland; 5 Nottingham University, Nottingham, UK; 6 Evolutionary Biology Group, Faculty of Biology, Adam Mickiewicz University, Poznań, Poland; 7 Faculty of Biology, Jagiellonian University, Kraków, Poland; 8 University of Jyvaskyla, Jyvaskyla, Finland

**Keywords:** Rodents, Bank vole, *Myodes glareolus*, Model organism, Genetics, Ecology, Parasitology

## Abstract

Rodents constitute a significant proportion of mammalian diversity, with their adaptability and wide distribution making them indispensable study organisms across various biological disciplines. While the laboratory mouse remains a predominant model rodent, the bank vole (*Clethrionomys glareolus*) offers a unique perspective as a wild rodent within the large subfamily Arvicolinae. Recognized for its relevance to studynatural ecology, the bank vole provides insights into complex ecological interactions, evolutionary adaptations, and disease dynamics. Despite recent recognition of its importance in specific research areas, there is a lack of a comprehensive and up-to-date exploration of its role as a model organism. This review addresses this gap by offering a holistic examination of the bank vole’s applications in ecology, evolution, biogeography, disease dynamics, and host–pathogen interactions. We emphasize novel insights into genetic variation, adaptation to climate change, population dynamics, experimental evolution, host-parasite co-evolution, and disease dynamics studies. By consolidating diverse research findings, this review provides a unique and comprehensive perspective on the bank vole’s contributions to understanding ecology and evolution, underscoring its importance as a model organism in shaping future biological research.

## Introduction

Rodents, comprising 2641 species, which make up 40% of all mammal species (number of species: 6581), play a central role in ecosystems worldwide, except Antarctica [101, 102]. Among them, the families Muridae (true mice and rats, including gerbils; 862 species) and Cricetidae (true hamsters, voles, lemmings, and New World rats and mice; 844 species) are the most diverse [125]. Their adaptability and wide distribution have made rodents indispensable study organisms in various biological disciplines such as ecology, behaviour [199, 229], genetics [174], drug screening and medical research [151, 153, 169, 187].

While the laboratory mouse (*Mus musculus*) remains a quintessential model rodent [20, 196], the bank vole (*Clethrionomys glareolus*) provides a valuable wild-rodent model from the subfamily Arvicolinae, which has a wide geographical distribution [115, 190].

Although *M. musculus* has also been studied in natural populations [130], its use as a model system historically stems from laboratory-based research and the early development of genetic tools–resources primarily aimed at biomedical and genetic studies. In contrast, the bank vole was not selected based on existing infrastructure or convenience, but because its natural history traits make it particularly well-suited for investigating ecological and evolutionary processes. Genetic and genomic resources for the bank vole have been developed specifically to support such integrative, question-driven research across both field and laboratory settings [117].

Recent reviews have underscored the bank vole's significance as a model organism in particular research areas. These include specific phenomena such as Puumala orthohantavirus (PUUV) [215], hepacivirus infection [177], parasites and ecoimmunology during biological invasions [142], prion disease [58], and adaptive phylogeography [111].

Although several reviews have addressed the bank vole in specific research contexts, a unified, cross-disciplinary synthesis remains lacking that draws connections across fields and highlights the broader value of this species as a model organism. The gap lies not in the absence of data, but in the absence of integration—bringing together knowledge from genetics, ecology, epidemiology, and evolutionary biology to understand the full potential of the bank vole system. This review addresses that need by consolidating current findings, identifying underexplored areas, and proposing future research directions, including how methodologies from other model organisms might be adapted to strengthen bank vole studies. By synthesizing the bank vole’s diverse applications in ecology, evolution, biogeography, disease dynamics, and host-pathogen interactions, this review serves as a one-stop resource for researchers and highlights the species’ unique contributions to biological research.

## Biology of bank voles

The nomenclature of bank voles has undergone several changes over recent decades, with the species temporarily referred to under the genus name *Myodes* before it was determined that the long-used name *Clethrionomys* was indeed correct. Consequently, in this review, we adhere to the usage of *Clethrionomys glareolus*, as recommended by the Mammal Diversity Database [214]. For a comprehensive discussion of the nomenclatural issues, please consult Kryštufek et al. [116].

**Fig. 1:**
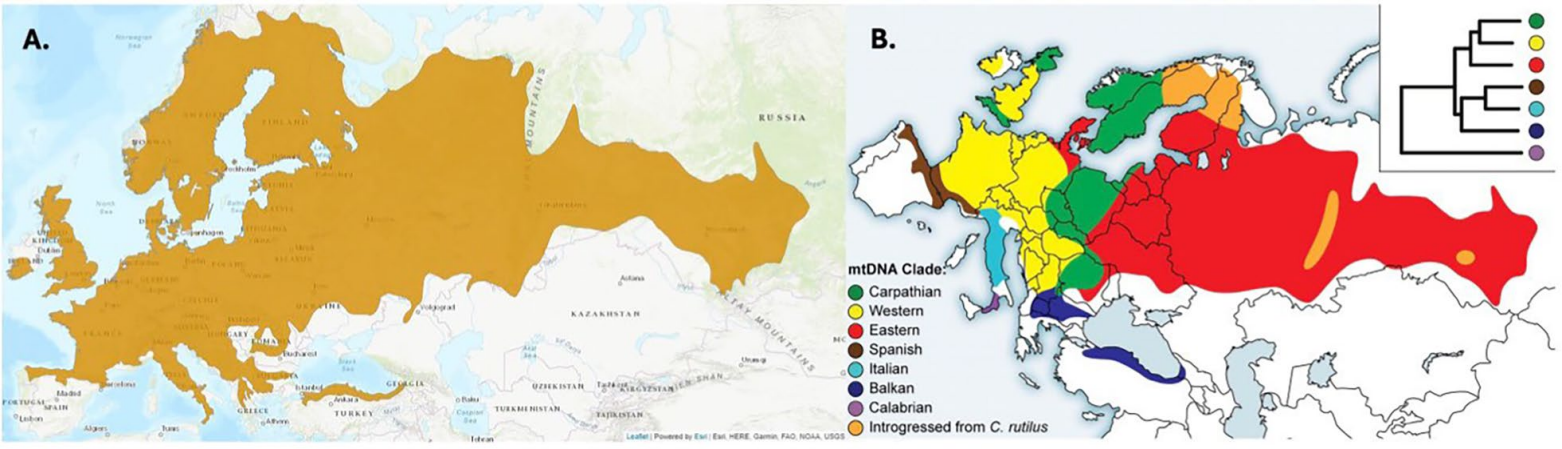
**A** The geographical range for *Clethrionomys glareolus* [82]. **B** Geographic distribution of bank vole phylogeographic clades based on mitochondrial (mt) DNA sequences, with a simplified phylogenetic tree in the top right inset. Adopted and reproduced from [111]

Bank voles are widely distributed and highly abundant throughout Western, Eastern, Northern and Central Europe [111]. Bank voles can be found at sites ranging from sea level to as high as 2400 m in mountainous terrain [195], depending on the geographic region. However, they are absent from the Mediterranean islands and much of Iberia (Fig. 1). Notably, bank voles were previously absent from Ireland (Stenseth, 1985), but were introduced inadvertently, likely from Germany, and are now spreading throughout the island as an invasive species [204]. It was first recorded in 1964 in County Kerry. Genetic and historical evidence suggest its introduction was accidental, likely via human activity. Since then, it has expanded its range considerably and is now well established in parts of the southwest. Its presence in Ireland offers a unique opportunity to study biological invasions, population expansion, and host-parasite dynamics in a relatively recent and well-documented context [154, 204].

The bank vole is currently classified as a species of Least Concern on the IUCN Red List of Threatened Species [80]. This designation reflects its wide distribution, large population size, and stable population trend across much of its range. Its broad ecological tolerance and adaptability further support its suitability as a model organism for studies in natural and semi-natural environments.

**Fig. 2:**
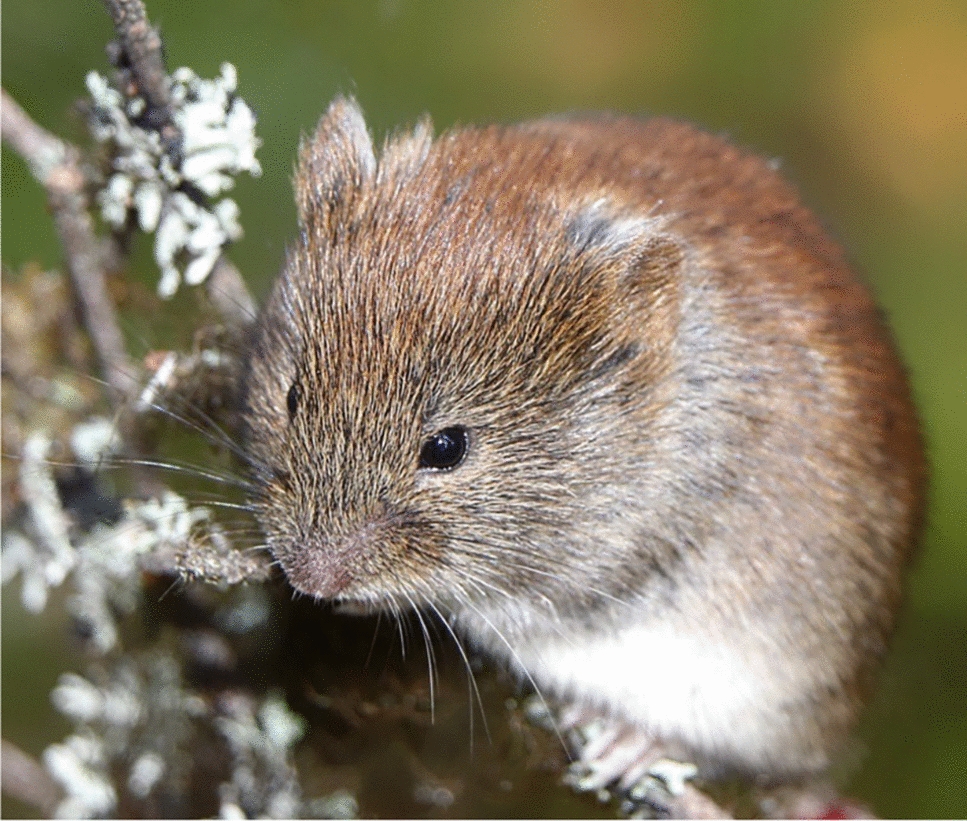
Bank voles have typical ginger-reddish and grey fur on their upper body and cream fur underneath. During the summer/autumn season, when population densities are high, they can often be spotted in the forests. Picture by Heikki Henttonen

The bank vole has a compact body with reddish-brown to greyish fur on the back and pale cream fur on the underside. Its snout is rounded, and its small ears are partially concealed by fur, though they are typically more visible than in *Microtus* species. The tail is relatively short—shorter than that of most mice but longer than in typical *Microtus* voles [117]. Because of its size and coloration, it can sometimes be mistaken for a large mouse. Young individuals up to 5–6 weeks old have darker juvenile fur, with more grey underparts [190] (Figs. 2, 4). Adult body length ranges from 8.3 to 12.1 cm, with a tail length of 3.5 to 6 cm. The weight of breeding *C. glareolus *typically ranges between 18 and 40 g [16, 127].

**Fig. 3:**
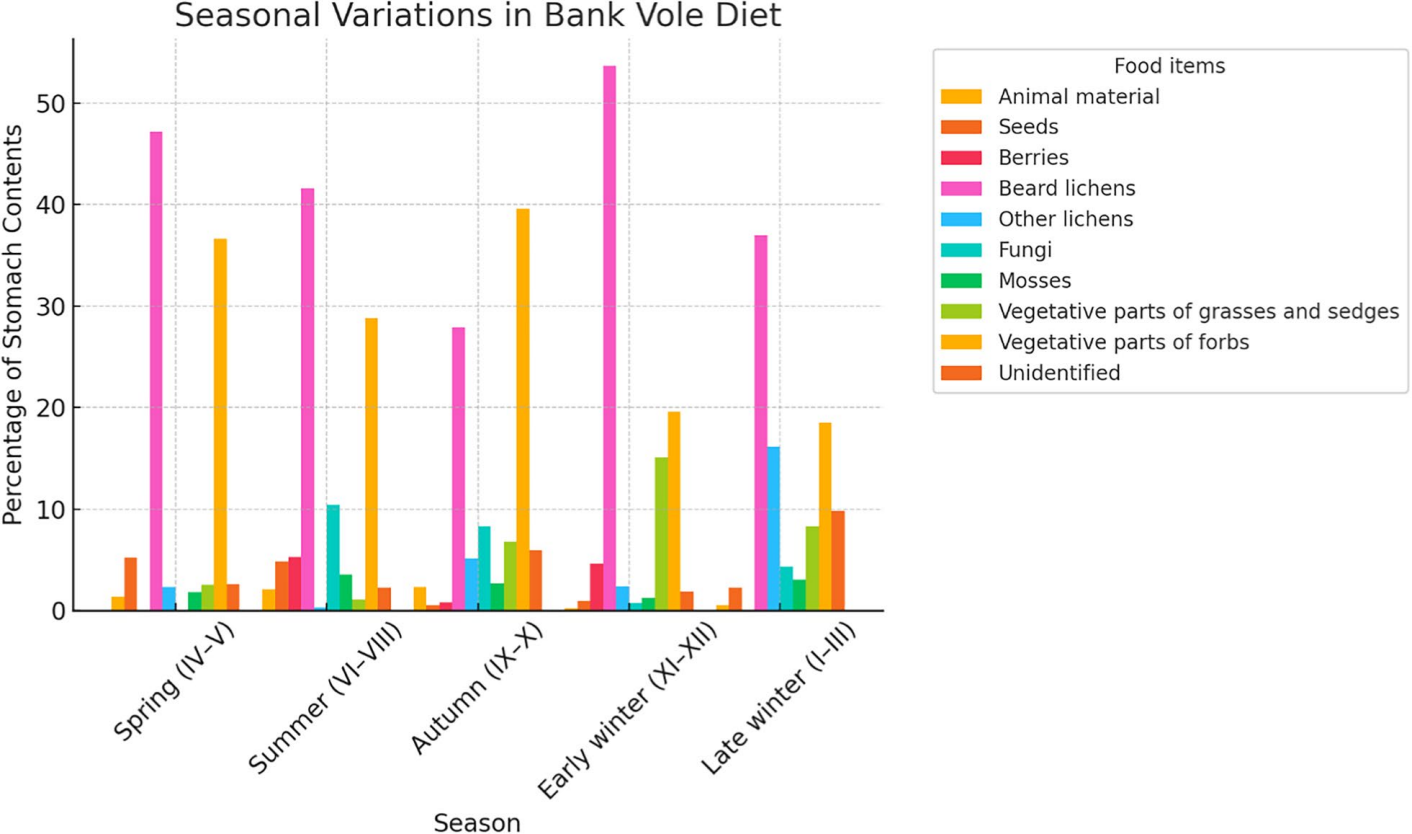
Seasonal variation in the diet of bank voles. Based on Viro and Sulkava [219] and Watts [225]

Bank voles are active day and night but exhibit a crepuscular peak activity. However, during the summer, their activity shifts primarily to nocturnal, although there is variation between geographic locations due to variations in local periodicity (e.g., Lapland in Finland). Bank voles do not hibernate during winter, storing food underground to ensure an adequate caloric supply [42]. These small rodents form long, branched burrows with many exits, creating tunnels under the leaf litter. Bank voles are mainly herbivores, feeding on the vegetative parts of herbaceous plants, berries, seeds, the bark of woody plants and fruits, but their diet is also enriched with insects, worms, and other invertebrates when they encounter them (Fig. 3) [52, 225]. Eating insects is the best way to meet the protein supply during the breeding and lactation periods, when protein requirements are greatest [24, 42].

Bank voles are largely herbivorous with 1–16% animal matter, 20–40% seeds and 40–50% green leaf material, depending on the region [191]. Fungi play a significant role in the bank vole diet in Northern Europe. Bank voles climb trees to collect arboreal lichens.

Bank vole populations exhibit both seasonal and multiannual fluctuations in abundance, each driven by distinct ecological mechanisms [97]. Seasonal cycles refer to predictable, within-year changes in population density. Typically, bank vole numbers are lowest in early spring due to winter mortality. As the breeding season progresses through spring and summer, populations increase, peaking in late summer or early autumn. This pattern is influenced by food availability, predation pressure, and reproductive rates. Multiannual cycles, on the other hand, are characterised by population peaks and crashes occurring over periods of 3–5 years. These longer-term fluctuations are driven by a combination of intrinsic factors, like social behaviour and reproductive strategies, and extrinsic factors, such as predation and food supply. For instance, during the increase phase of a cycle, higher social tolerance among females can lead to increased reproduction, while the crash phase may result from heightened predation and resource scarcity. Typically, in the temperate zone, masting years (good seed crops of beech, oak, etc.) impact bank voles' survival and breeding. On the other hand, in the snowy boreal zone in northern Europe, bank vole dynamics, sometimes called cycles, are driven by predation, particularly by specialists such as small mustelids [63, 69, 108]. In the temperate zone, the question is one of bottom-up processes, whereas in the boreal zone, it is top-down processes. Superficially, these vole dynamics may look similar, but the underlying causes are different. Understanding these dynamics is crucial for ecological studies and for predicting potential outbreaks of rodent-borne diseases (see Puumala orthohantavirus) [176]. Bank voles live mainly in woodland habitats but occasionally they migrate into parks, farmland, urban areas and clear-cuts due to forest management. They are not often found in large open grasslands where they would have to compete with the grassland specialists such as *Microtus agrestis* and *M. arvalis* (i.e. Koivisto et al. [104]) and because of the likelihood of being taken by predators [195].

Breeding female bank voles show strong territorial behaviour linked to their reproductive strategies [22, 43, 136]. The social organisation is gynocentric. The home ranges of breeding males are much larger than the territories of breeding females. The home ranges of breeding males overlap with the territories of breeding females [22]. Bujalska concentrated on breeding females and proposed a hypothesis in which territorial behaviour was suggested to function primarily for the protection and safety of the nestlings [23], e.g. against infanticidal conspecifics. However, Ostfeld [164] proposed an alternative model based on territoriality being determined mainly by food's spatial and temporal distribution [168]. Moreover, Koskela et al. suggested that the two hypotheses are not necessarily mutually exclusive, providing evidence for and against both hypotheses [109]. When the space available for territories during the breeding season becomes saturated, young maturing bank voles disperse, or they delay the maturation to the next year and remain as nonbreeding, nonterritorial subadults, which can stay in the territories/home ranges of the breeding voles. Prévot-Julliard et al. [171] analysed whether this delayed maturation is due to optimal decision or social constraints and found no support for the first alternative [170]. Experiments with supplemental food have shown that due to extra food territory size declines and a higher proportion of young females can mature, both increasing the density [231].

Bank voles exhibit three main dispersal periods throughout the year [44]:

1. Spring (maturation dispersal): Overwintered subadult voles, which do not breed during winter, initially occupy small home ranges. As they mature in spring, the territories of breeding females and the home ranges of breeding males expand. Individuals that fail to secure a territory are forced to emigrate.
2. Summer (juvenile dispersal): Juveniles begin to disperse as they mature. However, the rate of maturation can vary substantially between years, largely depending on the spring population density of overwintered adults.
3. Late autumn to early winter (weather-driven dispersal): Fluctuating snow conditions, melting and refreezing, can prompt additional dispersal in subadults preparing to overwinter.

Eccard and Ylönen [36] investigated the initiation of breeding following winter and found that while supplemental food accelerated breeding onset, local population density had a more significant regulatory effect. Thus, even under similar environmental conditions, populations with different local densities may exhibit non-synchronous breeding patterns [36].

The breeding season occurs from April to September. During mast years in temperate Europe, winter breeding is possible. There may be up to four litters annually, with the duration of pregnancy averaging 21 days, but occasionally longer, notably when females are still lactating [55]. The litter size can range from 1 to 6 in nature. Gustafsson et al. found that the number of pups depended on the number of previous pregnancies and reported a litter size varying from 4.3 to 5.3 pups per pregnancy [54]. Sometimes, males kill a female’s current offspring before copulation to ensure that their own offspring are advantaged [158]. The breeding strategies of bank voles under laboratory conditions have been comprehensively described in several foundational studies. Buchalczyk [21] provided an early and detailed account of reproduction in captivity, describing mating behaviour, gestation, and early development of bank vole pups. Gustafsson et al. [55] compared reproduction between colonies established from cyclic and non-cyclic populations. They found that voles from cyclic populations were generally larger, had larger litters, and exhibited faster growth in offspring, but suffered from higher offspring mortality and longer intervals between litters. Nyholm and Meurling [157] investigated reproductive activity in natural populations of bank voles from northern and southern Sweden. In cyclic northern populations, they observed longer breeding seasons and larger litters during population increase phases, but impaired reproduction during peak phases. In contrast, southern populations with no cyclicity showed less variation in reproduction, and early-season voles regularly matured within their first summer. Hansson and Henttonen [63] further investigated potential geographic patterns in reproduction and found no significant south–north differences in litter size across bank vole populations, suggesting that reproductive output was not a key factor explaining population cyclicity.

Population crashes are more severe in northern regions, where cyclic dynamics are most pronounced. In these northern areas, minimum densities during crash phases can drop to as low as one vole per 10 hectares or less, while peak densities may reach around 50–60 individuals per hectare. In contrast, in temperate zones, population densities also fluctuate but with lower amplitude: maxima range from 6–12 up to 50–100 individuals per hectare. The greater fluctuation in the north is driven not by higher peak densities, but by the extremely low minimum densities during population crashes [61, 69].

Since bank voles are active all year round, they constitute a quality food source for predators, including red foxes (*Vulpes vulpes*) [47], stoat (*Mustela erminea*) [141] and least weasel (*M. nivalis*) [167], many avian predators like common kestrels (*Falco tinnunculus*) and several owl species [193]. Geographic patterns in bank vole dynamics have been referred to as biomic differences in the community structure of specialist and generalist predators and their alternative prey or its absence [60, 63] Even if the biomic differences in population dynamics are clear some surprising changes can also occur within a biome. The strong cycles in north boreal Fennoscandia disappeared in the middle 1980’s, and they returned between 2010 and 2011 [32, 69, 71, 74]. The disappearance and return of cycles occurred simultaneously in a large area, which might suggest some climatic drivers.

## Trapping and laboratory maintanance

**Fig. 4:**
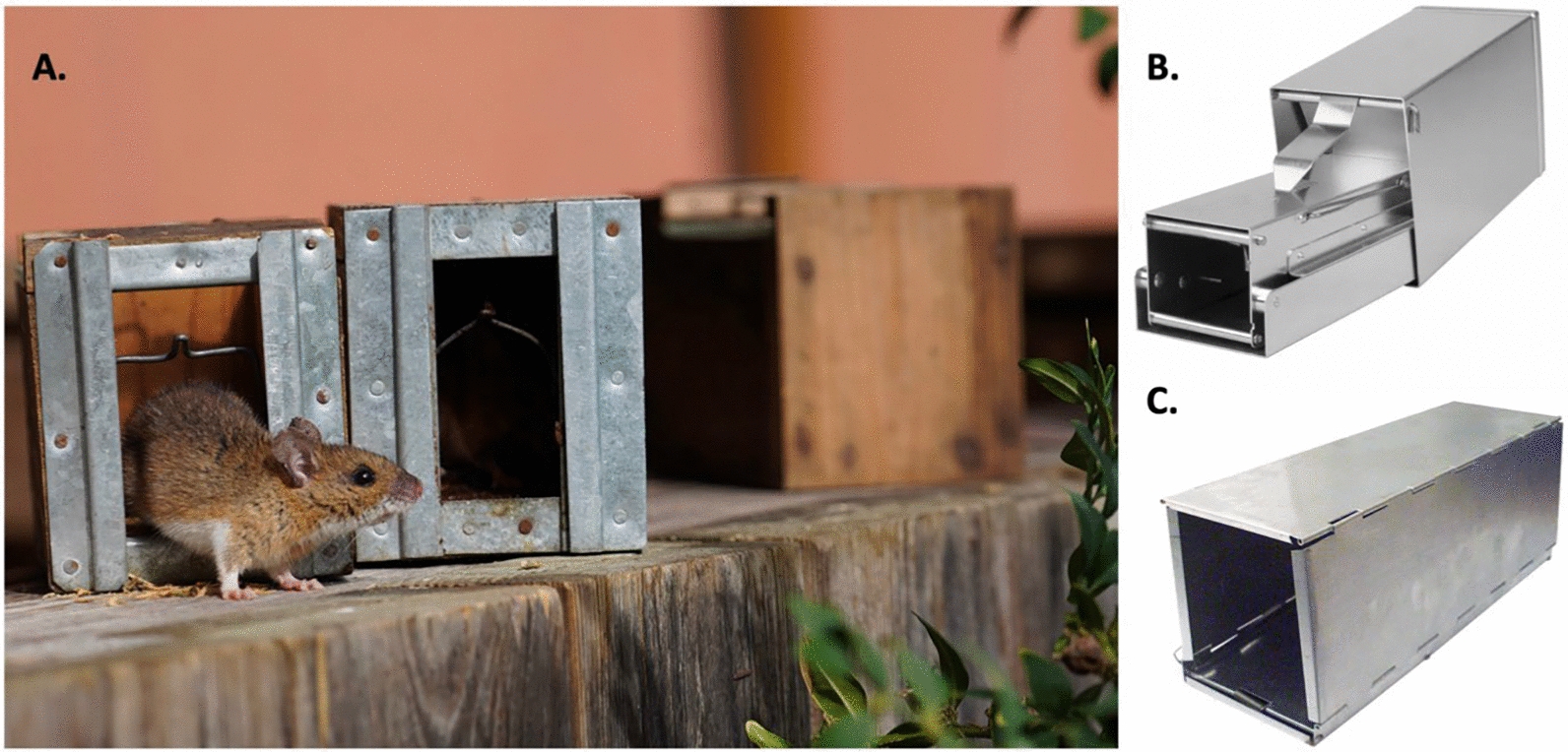
**A** Housemade wooden trap contains a small metal platform internally, which, when triggered, causes a metal door to close (image shows a wood mouse—*Apodemus sylvaticus*). **B** Longworth trap; **C** Sherman trap

Trapping bank voles in forested environments is a fundamental method in ecological and epidemiological research, used to estimate population density, monitor disease prevalence, and collect individuals for further physiological or genetic analysis. Live traps are designed to safely capture small mammals without injury, typically containing a bait compartment and a trigger mechanism that closes the trap door when the animal enters. A variety of bait types are used to attract voles, most commonly seeds (e.g., wheat, oats, sunflower), peanut butter, and fresh vegetables like carrot slices. Standardised live-trapping protocols have been developed and refined by various research groups (e.g., [16, 19, 50]), but are often adapted to local conditions and specific study goals. Depending on the research objectives, trapping sessions typically last 3–4 consecutive days and are conducted in deciduous or mixed forest habitats. Researchers employ a range of live-trap types, including plastic or wooden homemade traps, as well as commercially available Sherman, Longworth traps (Fig. 4) and “multiple-capture” Ugglan traps.

Typically, around 100–300 baited traps are deployed each night, using attractants such as wheat, oat or sunflower seeds, peanut butter, and carrot slices among others. Traps are arranged in lines, spaced 5–15 m apart, often with two traps placed within 2–3 m at each trap point to increase capture success [14, 16, 51]. This design allows for effective spatial coverage of the study area and maximizes capture rates. Captured animals are processed following established handling protocols, with data recorded on sex and age class, determined through morphological features such as body weight, molting patterns, and visible sexual characteristics. Age classification in bank voles typically follows a three-class system, based on morphological features such as body weight, molting patterns, and visible sexual characteristics. These classes, while widely used, are approximate and may vary between geographical regions due to environmental and genetic influences on growth and development. Following the methods of Behnke et al. [16], Grzybek et al. [51] and Loxton et al. [127], age classes can be established using principal components analysis of morphological traits—including dried eye lens weight and body mass—into: class 1 (immature juveniles), class 2 (mostly young adults), and class 3 (older, breeding individuals). This classification provides a practical framework for field studies, though it should be interpreted cautiously, especially when comparing populations across different habitats or regions.

Bank voles have been successfully maintained and bred under laboratory conditions in several studies. For example, [185] 185] housed animals in standard plastic mouse cages with sawdust bedding, at a constant temperature of approximately 20 ± 1 °C and a photoperiod of 16 light:8 dark. Although some laboratories adopt environmental parameters similar to those used for laboratory mice (e.g., ~ 21 °C, ~ 60% humidity, 12:12 h light–dark cycle), these are not necessarily optimal or required for bank voles. Researchers should consider both animal welfare and practical feasibility when designing husbandry protocols. Flexibility in environmental settings, such as using natural light regimes, broader temperature ranges, or less intensive humidity control, may be acceptable, provided they align with the species’ biology and ethical standards [62, 182].

Polycarbonate or polyethene cages should be lined with wood shavings and supplemented with nesting materials such as unbleached cellulose pulp, paper strips, and enrichment elements like cardboard tunnels to support natural behaviours and reduce stress [55]. Bank voles are generally fed standard pelleted rodent chow with water provided ad libitum, though fresh vegetables and seeds should be added to encourage natural foraging [79].

Social housing practices should account for sex and age to minimise aggression, particularly among breeding males. Breeding pairs should be monitored to ensure compatibility and reproductive success. Breeding typically involves keeping a male and female together for several days, followed by separation to reduce stress during gestation. Females give birth after a gestation period of 18–21 days, with litters averaging 3–7 pups. Pups are typically weaned at 20–24 days of age [81, 137]. However, when pairs are maintained permanently to produce multiple litters, to avoid the interference between voles from the previous litter and newborns, the litters can be successfully weaned also at day 17 [182].

Ensuring the safety of personnel involved in breeding bank voles in the laboratory necessitates a comprehensive occupational health and safety program. This program should encompass appropriate personal protective equipment (PPE), effective ventilation systems, and relevant vaccinations to mitigate potential health risks associated with animal handling (e.g. Puumala orthohantavirus) [155, 218]. This includes immunisations against tetanus and, where applicable, other zoonotic diseases, e.g. tick-borne encephalitis virus.

## Genetic and genomic foundations

In contemporary research, the development of a species as a model organism is strongly supported by the availability of genetic and genomic resources [2]. Advances in DNA sequencing technologies have facilitated the development of these resources even for non-traditional models like the bank vole. Although genomic resources for the bank vole are still emerging, recent progress has already provided valuable insights into its evolutionary history, immune adaptations, and physiological traits. The following sections highlight key genomic resources developed for the bank vole and illustrate their application across various biological contexts.

### Genomic resources: current status and challenges

Genomic resources for the bank vole remain limited compared to traditional model species like the laboratory mouse, reflecting the challenges of producing high-quality assemblies for wild species. Five fragmented genome assemblies are publicly available in GenBank, including one designated as the official reference genome (RefSeq accession: GCF_902806755.1). Although these scaffold-level assemblies lack chromosome resolution, they have supported single-nucleotide polymorphism (SNP) identification and evolutionary and developmental biology research [25]. Recently, a chromosome-level genome assembly with substantially improved contiguity and quality was produced and has been used for SNP analysis [139] and in transcriptomic studies of hantavirus-infected bank vole cells [41]. While Gallo et al. [41] include a brief description of the assembly, a full technical report and annotation are still in preparation.

Several reference transcriptomes further strengthen genomic resources. Babik et al. [7] assembled the first bank vole transcriptome from heart tissue, identifying gene expression patterns and SNP variation relevant to experimental laboratory evolution [7, 182]. Kotlík et al. subsequently sequenced and assembled a spleen transcriptome, aimed at SNP calling in population genomic studies [112], while Migalska et al. [144] characterised immune-related genes, emphasising the role of transcriptomic data in understanding rapidly evolving gene families [144].

Although genomic resources for the bank vole are less developed than for *M. musculus*, ongoing advancements are enhancing its value as an emerging model in ecology, evolution, and host–pathogen interactions. While progress has been slower due to fewer dedicated initiatives, expanding datasets and improved genome assemblies continue to strengthen the bank vole’s role in comparative and functional genomics.

### Comparative genomic insights

Preliminary comparative analyses reveal notable genomic distinctions between the bank vole and *M. musculus*. While their genome sizes are similar (~ 2.5 Gb; ~ 20,000 protein-coding genes), karyotypic studies indicate substantial differences in chromosome number and structure, likely affecting recombination rates, gene regulation, and evolution [5, 25]

A key feature of the bank vole genome is the expansion of repetitive DNA elements, including ribosomal DNA (rDNA), linked to increased genomic plasticity in response to environmental stress [87]. Such structural variability may contribute to adaptive flexibility, particularly in heterogeneous environments.

Distinctive patterns also appear in immune-related gene families. Comparative analyses suggest major histocompatibility complex (MHC) expansions, likely driven by pathogen diversity [6, 144]. While *M. musculus*—including wild-derived strains—exhibits considerable MHC polymorphism, domesticated strains often show reduced diversity due to relaxed selection [29]. The importance of immune gene variability, particularly at the MHC, is well established in the context of evolutionary ecology and conservation, and extensive MHC diversity in the bank vole likely reflects strong pathogen-driven selection, underscoring the role of immune adaptation in ecological success [194].

Sensory adaptations further distinguish the bank vole. Studies have identified novel odorant-binding proteins (OBPs) [126, 200], suggesting species-specific expansions that enhance chemosensory perception. Given that bank voles inhabit diverse environments and exhibit strict female territoriality during breeding [109], olfactory adaptations likely play a critical role in navigation, resource detection, mate selection, and communication.

The bank vole’s globin gene repertoire is unique in containing three functional copies of the α-globin gene (HBA-T1, HBA-T2, HBA-T3), a triplicate arrangement that differs from *M. musculus* but resembles that found in some other rodent species [140]. This variation may contribute to physiological adaptations in oxygen transport and metabolism, relevant for hypoxia tolerance and temperature fluctuations [202].

These genomic insights reinforce the bank vole’s value as a model for adaptation studies, including genome evolution, immune defence, and responses to environmental change. While *M. musculus* remains central to biomedical and genetic research, the bank vole offers a complementary perspective on genome evolution in wild, dynamically evolving species.

### Genetic structure, phylogeography, and hybridization

The bank vole exhibits a complex evolutionary history and genetic structuring of natural populations, shaped by past climatic fluctuations, multiple glacial refugia, and postglacial expansion dynamics. Early mitochondrial DNA (mtDNA) studies revealed distinct genetic lineages across Europe (Fig. 1), each associated with different glacial refugia, with a key role for “cryptic” refugia in the Carpathians [110]. More recent genome-wide SNP studies have refined these findings, revealing intricate patterns of postglacial expansion and secondary contact zones, particularly in Central and Northern Europe [76, 138].

The “Celtic fringe” pattern in British bank voles is one of the best-documented phylogeographic examples in mammals [189]. It refers to the persistence of an early postglacial genetic lineage in the northern fringe of Britain, which was later largely replaced across most of the island by a second wave of colonizers from continental Europe (Fig. 1). Initially detected through mtDNA phylogeography [189] and later substantiated by genome-wide SNP analyses [112], this pattern exemplifies the role of population replacement in shaping genetic diversity and demonstrates how genomic data can distinguish between population processes and single-locus patterns. In contrast to Britain, bank voles in Ireland were introduced by humans. Mitochondrial DNA analyses show an affinity with German populations, aligning with historical evidence that the species was introduced from Germany in the 1920s [204].

Hybridization has also shaped the bank vole genome. In northern Fennoscandia and Russia, extensive historical mitochondrial introgression from the northern red vole *Clethrionomys rutilus* has been documented (Fig. 1) [1, 207], yet nuclear genomic analyses indicate limited overall gene flow [138]. This suggests adaptive retention of introgressed mtDNA rather than widespread hybridization [17]. These findings highlight the role of historical admixture in shaping the bank vole’s genetic diversity, potentially affecting its ecological and physiological adaptations.

With its rich phylogeographic data, the bank vole provides a powerful model for understanding evolutionary responses to range shifts, secondary contact, and selection pressures in changing environments.

### Genomic adaptation to climate change

Understanding genetic adaptation mechanisms is increasingly crucial as climate change continues to alter habitats and species distributions. The bank vole provides a unique model for studying genome-environment interactions, particularly in the context of physiological adaptation to temperature fluctuations.

One of the best-studied adaptive traits in the bank vole is haemoglobin (Hb) polymorphism [56, 113, 203]. Research on British populations has demonstrated that distinct Hb variants are distributed along temperature gradients, with evidence suggesting selection for alleles that enhance oxidative stress resistance under warmer climatic conditions [113, 139]. This system provides a tractable model for understanding how standing genetic variation facilitates climate-driven adaptation [37].

Beyond haemoglobin variation, whole-genome sequencing has identified climate-adaptive SNPs in genes involved in cellular stress response, which appear to be under selection in populations inhabiting warmer or more seasonal environments [139]. This suggests an adaptive advantage in coping with climatic fluctuations. Interestingly, some of these genes also contribute to hypoxia tolerance in high-altitude mammals, including humans, highlighting their broader evolutionary role in environmental adaptation [139]. These findings indicate that shared genetic pathways may underlie adaptation to both warming climates and oxygen-limited environments.

Peripheral populations, such as those in Britain, may be approaching adaptive limits due to reduced genetic diversity, constraining their ability to respond to future climate change [139]. This raises concerns about local population viability and underscores the importance of maintaining population connectivity and facilitating gene flow for long-term species persistence [75]

By integrating genomic, physiological, and ecological data, bank vole studies provide valuable insights into climate adaptation mechanisms, with broader implications for understanding evolutionary responses to rapid environmental change. These findings also have potential applications in conservation biology, particularly in predicting population resilience and informing strategies to mitigate biodiversity loss under future climate scenarios.

## A laboratory model of adaptive radiation

Experimental evolution under controlled laboratory conditions offers a promising tool for cross-validating conclusions and testing hypotheses concerning the evolution of behavioural and morpho-physiological adaptations, and a basis for studying neurobiological, biochemical, and molecular mechanisms underlying the adaptations observed at the organismal level [201]. In recent decades, the value of and the need for such experiments have been recognised. However, the selection experiments on mammalian models are still scarce, and most of them have been performed on laboratory strains of mice or rats [72]. Conducting long-term selection experiments on mammalian models presents several logistical and practical challenges. These include the need for sustained funding over multiple generations, which span several years, and securing and maintaining specialised facilities that meet strict animal welfare standards [223]. Ethical approval processes for such studies are often complex and must ensure the well-being of animals over extended periods, adding another layer of responsibility and planning [98]. In addition to the fact that such an experiment must be extended over years, the most challenging aspect is that such experiments require keeping a large number of animals and making measurements on many animals. This is because (a) each selection direction and unselected control should be represented by at least a few replicate lines to allow methodologically valid tests of the effects of selection [68], (b) to avoid inbreeding each line must be represented by at least 10 successfully breeding pairs (and therefore the number of mated pairs must be considerably larger), and (c) to allow effective selection each pair should produce at least 10 offspring. Several early selection experiments were unreplicated or had only two replicate lines. However, such experiments cannot reliably distinguish the random effects of genetic drift from the directional effects of selection [68]. The key point is that, in selection experiments, the experimental unit is a line (population), not an individual. In a typical study based on phenotypic manipulation, an experiment involving only three individuals in the treatment and control groups would hardly be considered adequate. Such an experiment could not demonstrate a statistically "significant" effect (*p*-value < 0.05) based on a nonparametric ranked test (the lowest possible *p*-value is 0.05). Thus, in selection experiments, it is advisable to include at least four replicate lines in each selection group. Consequently, with one selection direction and a control group, the colony should include nearly 1,000 animals in each generation. These factors help explain why such experiments remain relatively rare, particularly in wild-derived or non-traditional model species.

One of the few exceptions was an experiment on bank voles selected for high and low reproductive output [188]. However, the experiment was limited in scale (there were no replicate lines) and lasted for only a few generations. Moreover, the selected lines were used primarily as a basis for quantitative genetic analyses of reproductive traits, rather than as part of a broader research program exploring the effects of selection. Despite these limitations, the study provided important insights into life-history evolution in small mammals. The quantitative genetic analysis revealed that, although a phenotypic trade-off between the offspring number and size was observed (negative phenotypic correlation), this was not underpinned by a genetic trade-off (no negative additive genetic correlation). Instead, the negative phenotypic correlation was driven by negative correlation of the permanent and temporary environmental effects, while direct additive genetic correlations between these traits were neutral or even positive. The analysis also suggested a presence of negative correlation between direct genetic effects on the litter size and maternal genetic effects on body size. This suggests that in bank voles, the evolution of offspring number and size is not constrained by antagonistic direct genetic relationships, but rather shaped by complex interactions between direct and maternal genetic effects.

**Fig. 5:**
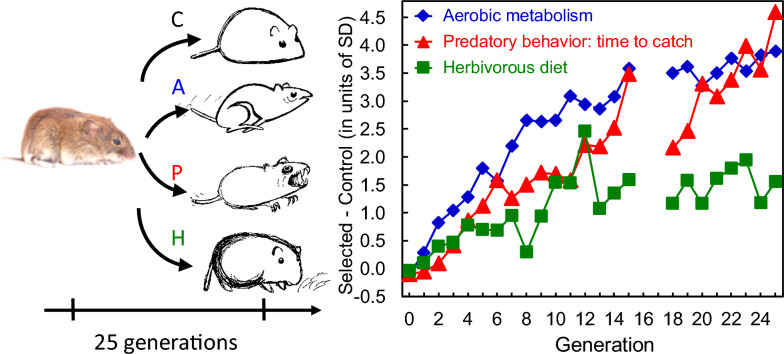
The design of the multidirectional artificial selection experiment on bank voles and the direct effects of selection, expressed as differences between the selected and control lines in the units of phenotypic standard deviation (a gap in generations 16–17 indicates a period of relaxed selection) [182], 185] (cartoons: January Weiner; photo: Maciej Grzybek)

Another selection experiment on bank voles (conducted in the Institute of Environmental Sciences of the Jagiellonian University in Kraków) is unique in its scale [182], [185]. The large laboratory colony was established from wild-trapped animals. Before launching the selection experiment, the voles were random bred for a few generations and served as a basis for quantitative genetic analyses of metabolic traits [181, 183]. Unlike in most other selection experiments on vertebrates, the voles were selected in three distinct directions: the increased rate of aerobic metabolism (Aerobic–A lines), the ability to grow on a herbivorous diet (H–Herbivorous lines), and the intensity of predatory behaviour (Predatory–P lines; Fig. 5). Four replicated lines in each of the selection directions and unselected control (C) were maintained. Thus, the experiment can be treated as a laboratory model of adaptive radiation. The direct effects of selection appeared already after two generations, and in generations 20–25 differences between the selected and control lines were of the order of 1.5 to 4 phenotypic standard deviations (Fig. 5).

This multidirectional selection experiment provides a unique model system for experimental evolution that can be used for a wide range of research concerning questions at all levels of biological organization, from the molecular to the ecological. One of the major aspects of the research program concerns the correlated evolution of behavioural and physiological performance traits. The distinctly selected lines showed different “personalities” in an open field test [131], though they did not differ markedly in hormonal stress response characteristics [121, 122, 124] or learning capability [28].

Compared to Control lines, Aerobic line voles evolved a 70% higher maximum swim-induced rate of oxygen consumption (the trait directly selected for). During the tests the animals were not forced to exercise; thus, the selection promotes both an increased aerobic capacity per se (which is measured in a forced-running test) and a propensity to exercise. Indeed, both components increased in the Aerobic lines. Thus, the lines have become a suitable model for studying both neurophysiological basis of the motivation to exercise [83–85] and the mechanistic basis of the differences in metabolic performance [86, 121, 198].

Similarly, in the Predatory lines, in which the selection criterion was a ranked time to capture crickets in four 10 min trials, about 85% of individuals captured crickets in at least one of the trials, while only about 15% of Control voles showed the predatory behaviour. However, selection also increased the swiftness of capturing crickets in the successful trials. Again, the lines provide a suitable model for the study of both the motivation to hunt and the neurological basis of hunting skills (work in progress).

Voles from the Herbivorous lines, selected for an ability to maintain body mass during a 4 day test with low-quality diet, lose approximately 2 g less mass than those from Control lines, and our preliminary analyses indicate that the difference arose because of both an increased readiness to eat the low-quality food, increased capacity to process the food, and a decreased locomotor activity. In recent decades, awareness of the importance of microbial symbionts has grown, leading to the development of the holobiome concept and hologenomic evolutionary theory [18]. An analysis of gut bacteria showed an altered microbiome community composition in the H lines [103, 123], which supports some assumptions of this theory.

Voles from the Aerobic lines offer a suitable model to investigate several aspects of physiology related to the rate of metabolism. They have increased basal and average daily metabolic rates, thermogenic capacity, and reproductive output, and are more vulnerable to overheating, but do not markedly differ in the ageing pattern, the degree of oxidative damage or reponses to high-energy diet [35, 48, 78, 160, 180, 184, 185, 197].

Selection experiments also provide a powerful tool to investigate molecular background of physiological and behavioural traits. Whole transcriptome analyses showed that the evolution of aerobic metabolism was mainly due to an altered level of expression of several genes, while changes in allele frequencies of SNP loci played a more significant role in the evolution of the predatory behaviour [106, 107]. Thus, the general “genetic architecture” underlying these traits appears to be distinct. The analyses also indicated several candidate genes that could contribute to the differences between the lines, which opens perspective for further genomic and molecular analyses (work in progress).

However, perhaps the most exciting perspective offered by the selection experiment on bank voles—rather than on laboratory mice—are experiments performed under natural or semi-natural conditions. The first such experiments showed that under semi-natural conditions, voles from the Predatory lines had a diet representing a higher trophic level, i.e., containing more animals, than those from the control lines [57], and voles from the Aerobic lines had an altered gut microbiota, even though no such difference was found under laboratory conditions [59].

The selection experiments not only provide suitable models to study various aspects of animal physiology and behaviour and biochemical and molecular mechanisms underlying these characteristics, but can also provide models of the processes in the natural evolutionary processes, in which the population suddenly becomes subject to some novel selection pressures. For example, the ongoing invasion of Ireland by the bank vole presents a compelling example of adaptive processes in a natural, expanding population [142]. It strengthens its value as a model organism beyond laboratory or enclosure studies. Introduced in the early twentieth century, bank voles have expanded at a consistent rate of approximately 2.5 km/year, offering a rare opportunity to study evolutionary dynamics in real time. Genomic analyses have revealed a decline in genetic diversity toward the range edge, alongside evidence of spatially consistent selection on loci related to immunity and behaviour—indicating adaptation during expansion. Additionally, spatially explicit modelling has shown that local habitat features and density-dependent dispersal behaviours shape the vole’s spread, consistent with a Type 1 range expansion [228]. Future selection experiments could be designed to mimick such processes under controlled laboratory conditions, and compare the selection effect for particualr traits with those observed under such “natural experiments” as the invasion of a new area.

## A model organism to study evolutionary immunogenetics

Bank voles have provided important insights into the evolution of the immune system under pressure from parasites. Much focus in evolutionary immunogenetic studies has been on the Major Histocompatibility Complex (MHC) genes, which encode proteins responsible for presenting antigens to T-cell receptors (TCRs), thereby eliciting adaptive immune responses. MHC molecules tend to specialise in the presentation of antigens from intracellular (class I) or extracellular (class II) pathogens. Bank voles were used to test a hypothesis explaining extreme polymorphism of MHC genes in vertebrates (with dozens to hundreds of allelic variants per locus found in populations).

Using the parasitological dataset of more than 900 bank voles, sampled within the long-term PolVole project in northeastern Poland (spanning 11 years), demonstrated how parasites tend to adapt to the most common MHC alleles [179]. The study thus provided support for one of the major hypotheses explaining the maintenance of MHC polymorphism, which is negative frequency-dependent selection resulting from fast-evolving pathogens adapting to common MHC alleles (reviewed in [173]).

Furthermore, bank voles were used to forward our understanding of the evolution of the number of MHC genes in an individual genome. Across vertebrates, individual genomes typically contain from a few to a couple of dozen MHC loci, which is only a fraction of the allelic diversity found in their populations. Raising the question of why natural selection appears to limit MHC gene number despite its potential benefits in antigen presentation.

The optimality hypothesis has been proposed to explain this limitation by a trade-off between the ability to present antigens from diverse pathogens and the loss of TCR diversity due to the autoimmunity-protection mechanism involving the deletion of self-reacting lymphocytes [156]. Consequently, excessive MHC diversity is hypothesised to increase the risk that antigens presented by MHC would not find a lymphocyte partner with an appropriate TCR, thus failing to elicit an appropriate immune response. Bank voles were well suited to test this hypothesis because of their considerable inter-individual variation in the number of expressed MHC genes (6–19 loci [146]), surpassing variation in inbred laboratory mice, which typically express only five classical MHC genes with little variation between individuals, and thus represent another valuable system for studying natural immunogenetic variation [120]. However, the hypothesis has proven difficult to test due to technical difficulties of genotyping paralogous MHC loci, as sequence similarity across loci precluded the design of locus-specific primers. Such co-amplifying MHC loci, observed across vertebrate taxa, were notoriously problematic for genotyping using classical methods. Immense TCR diversity, arising during somatic recombination, posed an even greater challenge. Bank voles were used as a first non-model system in which these technical challenges were overcome using a combination of high-throughput sequencing and exploiting bioinformatic developments [8, 145]. This allowed Migalska et al. [146] to demonstrate that indeed in bank voles the TCR repertoire correlated negatively with the number of MHC class I, but not MHC class II alleles, possibly due to additional pathways of dealing with auto-immunity available to CD4 lymphocytes interacting with the latter.

Bank voles are also an emerging model system for the study of host-parasite co-evolution at a gene level, focusing on the Lyme-disease spirochete, *Borrelia spp.* as an infectious agent. Bank voles are one of the major hosts of this tick-transmitted spirochete in Europe. There is evidence for fitness consequences of infection with *Borrelia* sp for bank voles [26]. Infection with *B. afzelii* in voles was found to be associated with TLRs in Swiss populations [211], although this finding was not confirmed in a Polish population of bank voles, or in a controlled laboratory experiment [45, 206]. This discrepancy may reflect differences in local host genetic backgrounds, pathogen strain variation, or ecological conditions, suggesting that immune gene-pathogen associations may be context-dependent. However, a set of other candidate genomic regions associated with susceptibility to *B. afzelii* infection was identified using reduced-representation genome sequencing [31].

The recent publication of the annotated bank vole genome promises a finer level of resolution. The advantage of using *Borrelia* is that the molecular basis for its infectivity is relatively well understood because of its biomedical importance. OspC is one of the outer subphase proteins of *Borrelia* that allow the spirochete to evade host immune responses. OspC is at the same time immunogenic, and recent work [172] suggested that strains carrying different OspC variants interact with MHC DQB (class II) in determining the success of infection. Such causality has been suggested by more recent work demonstrating that MHC determines the level of antibodies against specific OspC variants [179].

Anti-borrelial antibodies have been demonstrated to be transmitted from mother to pups in [46], and similar findings have been reported for antibodies against Puumala orthohantavirus [92]. This maternally derived immunity has significant implications for ecoimmunology, as it demonstrates that passive immunity can influence infection dynamics in early life stages, potentially affecting both individual fitness and pathogen transmission within wild populations. Studying such mechanisms in natural systems, such as the bank vole, provides valuable insight into how immune defences are transferred across generations and how they interact with environmental and evolutionary pressures.

## Eco-epidemiological studies

**Fig. 6:**
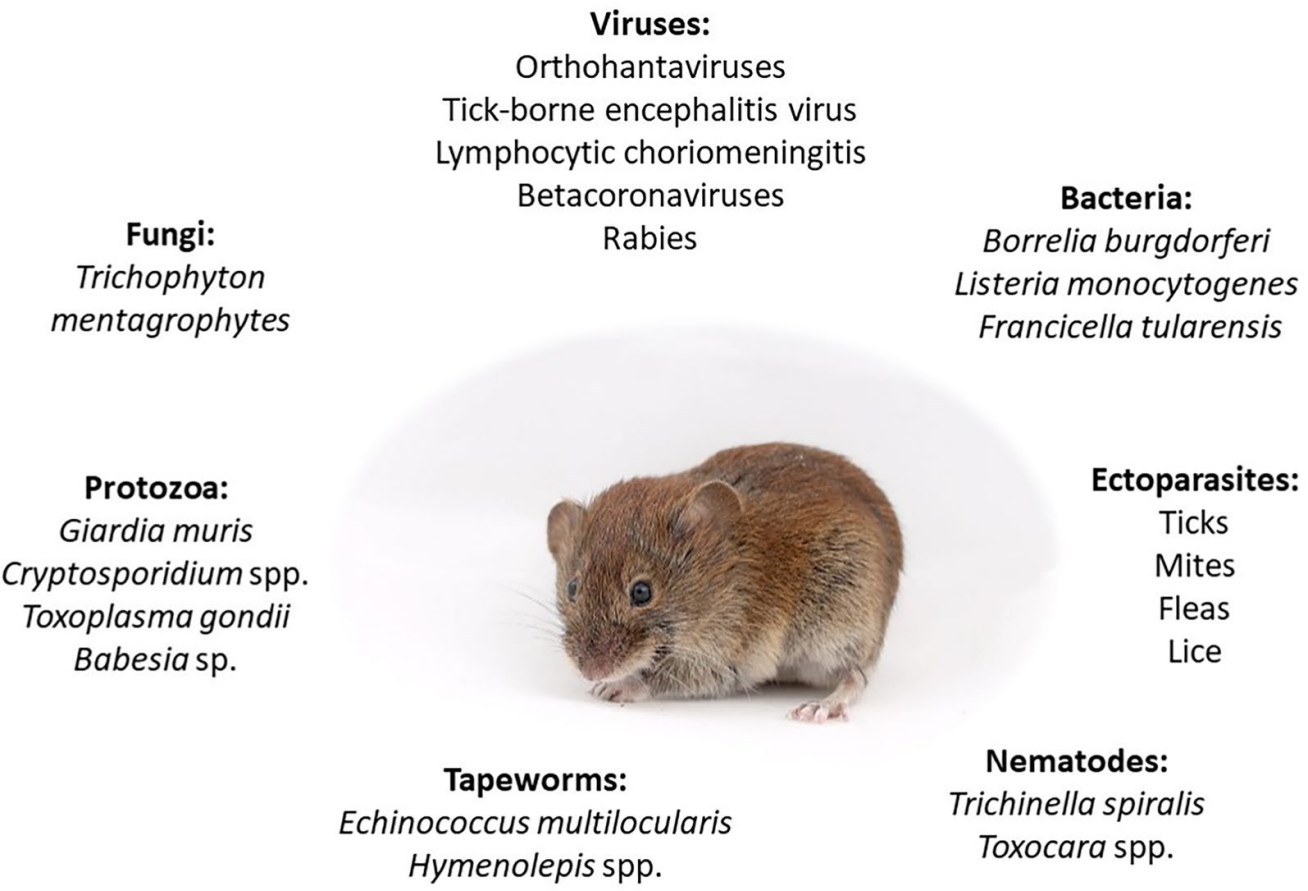
List of the most important zoonotic pathogens vectored/transmitted by bank voles (Picture: Maciej Grzybek)

Bank voles, as the key component of woodland rodent communities, act as an important reservoir of numerous pathogens (Fig. 6) including metazoan and protozoan parasites, bacteria, fungi, and viruses [101, 102]. The wide distribution of *C. glareolus* in the Palearctic and its high abundance throughout Europe explain why many helminthofaunistic studies have been conducted on this host [16, 65]. Their ecological flexibility and high population densities across diverse habitats contribute to their exposure to, and maintenance of, a wide range of infectious agents, including zoonotic viruses [52]. Due to their wide distribution in various environments, bank voles act as a reservoir for a range of different infectious agents [33, 215].

Numerous studies on bank vole parasites, including ectoparasites, helminths, haemoparasites, and intestinal protists, have been conducted in the Mazury Lake District of northeastern Poland, forming the basis of one of the most comprehensive long-term monitoring efforts in Central-Eastern Europe [15, 64, 166]. This initiative, known as the PolVole Project, began in 1999 and has involved repeated sampling in 1999, 2002, 2006, 2010, 2014, 2018, and 2022 [15, 16, 49, 51].

### Ectoparasites

The ectoparasite community of bank voles consists of juvenile ticks, mites, a range of flea species and lice [226] Bank voles play an important role in transmission of flea- and tick-borne pathogens because they serve as the source of infection for numerous generations of fleas and juvenile ticks, larvae and nymphs feeding on them. Bank voles serve as hosts for *Ixodes ricinus*, *Ixodes persulcatus*, *Ixodes trianguliceps* and *Dermacentor reticulatus* tick species [77, 129].

In a long-term study of the tick community of bank voles conducted in North-Eastern Poland, bank voles were infested mainly with the larvae of *I. ricinus*, with *I. ricinus* nymphs, and with nymphs and larvae of *D. reticulatus* [166]. They were also infested with several generalist species of fleas (C*tenophthalmus* spp, *Megabothris* spp., *Hystrichopsylla talpae*) [3, 88].

### Haemoparasites

In consequence, the community of blood parasites monitored during the long-term PolVole Project (1999, 2002, 2006, 2010, 2014) consisted of tick-borne pathogens—(*Babesia microti* [14], *Borrelia burgdorferi* sensu lato) [192], flea-borne parasites (*Hepatozoon erhardovae, Trypanosoma evotomys*) and bacteria (*Bartonella* spp.) [13, 14]. Zoonotic species and strains (genotypes) of *Bartonella* and *B. microti*, including *Bartonella grahamii* and *B. microti* Jena genotype, were identified in bank voles in the Mazury Lake District, indicating a possible risk for humans in the region [226]. Both extrinsic (year and season of study, site of the study) and intrinsic factors (especially, age of voles) had a significant effect on species richness, prevalence, and abundance of haemoparasites [14, 70, 165]. For example, *Bartonella* spp. infections in *C. glareolus* were more common during autumn months and in juvenile and young adults (age classes 1 and 2) than in adults (age class 3) [227].

### Intestinal protozoan parasites

An important component of the study on bank voles was determining the role of bank voles as a reservoir of intestinal protozoan parasites: *Cryptosporidium* and *Giardia* spp. [9, 10]. In an eight-year period, a relatively high prevalence of these intestinal parasites in three bank vole populations (54% for *Cryptosporidium* and 58% for *Giardia* spp.) was observed. An especially high prevalence of *Giardia* spp. was repeatedly observed in spring in overwintered voles (Bajer, unpublished). Preliminary molecular typing revealed the occurrence of zoonotic species/genotypes in naturally infected voles, including *Cryptosporidium parvum and Giardia intestinalis* (syn. *G. duodenalis*) [12]. Several *Cryptosporidium* strains have been found in voles, including bank voles, and shrews in Finland [11, 51, 66, 100]. The prevalence of intestinal protozoa was positively associated with nematode infection (*Heligmosomum mixtum, Heligmosomoides glareoli*), especially in the oldest voles (age class 3) [11]. This pattern of increasing parasite richness with host age is consistent with earlier findings from Finland, where Haukisalmi, Henttonen, and Tenora [66] showed that older voles harboured the most diverse helminth communities, with parasite dynamics influenced by host population cycles and parasite life histories. Grzybek et al. [51] further confirmed these trends in Polish populations, observing that mean nematode species richness increased with host age, and highlighted how seasonal and demographic factors contribute to parasite assemblage structure across individuals. Together, these studies provide a broader framework for understanding age-dependent patterns in helminth community dynamics across different vole populations and regions [66].

### Viruses

Bank voles serve as hosts for various viruses, including cowpox virus, Puumala orthohantavirus (PUUV), and lymphocytic choriomeningitis virus (LCMV). Many rodent-borne viral diseases are sustained in nature through direct transmission within and between rodent species, without the involvement of arthropod vectors, and viral infections cause persistent infections in rodents (e.g. PUUV). However, some others, such as Cowpox virus infections, last only a few weeks in rodent hosts [38]. Transmission occurs through contact with rodent body fluids or excreta [217]. Among the most prevalent rodent-borne zoonotic viruses carried by bank voles are hantaviruses, LCMV, Cowpox virus, and Ljungan virus (LV) [215]. Puumala virus (PUUV) is widespread in bank vole populations [89, 161]. Hantavirus infections in bank voles are chronic, resulting in life-long persistent viral replication [90, 93],). Consequently, the rodent host can remain infectious throughout its lifespan [143, 222].

Ribas Salvador et al. studied hantavirus–helminth coinfections in natural populations and found that interactions between helminth presence and landscape features can either enhance or reduce the likelihood of coinfections between Puumala orthohantavirus (PUUV) and *H. mixtum* or *Aonchotheca muris-sylvatici* [186]. Guivier et al. studied how environmental heterogeneity influences immune responses and susceptibility to PUUV infection in bank voles. By examining the expression of immune genes *Tnf-α* and *Mx2*, the researchers found that PUUV load was negatively correlated with gene expression, suggesting that stronger immune responses may suppress viral replication. Habitat structure, particularly forest fragmentation, was linked to variation in immune gene expression, with lower *Mx2* levels in large forests. Co-infection with the helminth *H. mixtum* also reduced proinflammatory responses, potentially increasing PUUV susceptibility. The findings highlight how landscape features and co-infections interact with host immunity to shape PUUV epidemiology [53]. These studies demonstrate that both helminth co-infections and environmental heterogeneity significantly influence host immune responses and the risk of Puumala hantavirus infection, providing key insights into the complex ecological drivers of coinfection dynamics in wild rodent populations. Cell lines derived from bank vole represent an important resource for studying virus-rodent host interaction and pathogenicity. For example, experimental infection of bank vole cells with three different orthohantaviruses has shown that they are restricted at different levels of the replication cycle, likely due to differences in the initiation of the innate immune response. Only PUUV achieves a complete cycle in bank vole cells, whereas *Microtus* carried Tula virus (TULV) does not enter cells and Prospect Hill virus (PHV) infects cells but does not produce infectious particles [41].

Bank voles, along with other rodents such as *Apodemus* spp. and *Microtus* spp. have also been considered reservoir hosts for Cowpox virus [27], the only known wildlife-borne Orthopoxvirus (OPV) in Europe [99]. However, the role of bank voles as Cowpox reservoirs was recently questioned by Franke et al. [40] based on experimental laboratory infections. However, field studies have concluded that since, bank voles show a high Cowpox seroprevalence, they most likely act as the reservoir for the maintenance of poxviruses [39, 52, 208]. Although isolating the virus from vole samples is rare, probably because earlier wrong tissues have been analysed, human Cowpox infections primarily occur through contact with diseased incidental hosts, such as cats or domestic rodents [27].

Recently, Wasberg et al. [224] highlighted the discovery of a novel Betacoronavirus isolated from *C. glareolus*. Consequently, scientists now think that various viral strains co-circulate within the bank vole population, but further analysis is required to comprehend the transmission pathways. Experiences of the COVID-19 pandemic emphasized the need for constant biomonitoring of possible virus hosts and reservoirs monitoring. Experimental studies showed that bank voles can be infected with the SARS-CoV-2 virus, however, transmission to contact animals has not been detected [213]. Bourrett et al., sampled 1202 rodents trapped across Europe, but found no SARS-CoV-2 seropositive bank voles [19]. Knowledge of most viral zoonotic infections in rodents is based on short-term cross-sectional studies reporting pathogen prevalence at a single location and time point. Nevertheless, some recent studies have been investigating the dynamics of orthohanta- and arenaviruses in wild rodents [39], over long periods and exploiting the resulting long-term datasets for detailed analysis at [52, 175, 209, 220]. Unlike cross-sectional studies, these long-term investigations have revealed more complex epidemiological patterns, including seasonal fluctuations in infection prevalence, delayed density-dependent transmission, and associations with environmental drivers such as mast seeding or climate variability. These findings highlight the importance of longitudinal monitoring for understanding the temporal ecology of rodent-borne viruses.

The contrasting biomic differences in population dynamics of bank voles are reflected, for example, in the epidemiology of Nephropathia Epidemica, caused by Puumala orthohantavirus [119, 162]. In temperate broad-leaf forests, heavy mast seeding (usually in beech or oak) during an “mast year” boosts overwinter survival, leading to a multi-annual population high that peaks seasonally in late spring–summer, when breeding activity is at its maximum. Consequently, the human disease peak in these regions also falls in summer [97]. By contrast, in the northern boreal zone most transmission among bank voles occurs in late autumn–winter among non-breeding subadults voles [220], and the human incidence peak is recorded from October to January. The virus remains infectious outside the host for up to 2 weeks at room temperature and even longer in winter’s low temperatures [91].

### Tick-borne encephalitis virus

The tick-borne encephalitis virus (TBEV), responsible for tick-borne encephalitis (TBE), is a zoonotic flavivirus in the Flaviviridae family that is endemic across the Northern Palearctic region, from Central and Northern Europe to Siberia and Japan in the Far East [118]. In nature, it is maintained in a cycle involving ticks of the *Ixodes persulcatus* and *Ixodes ricinus* complex as vectors and a range of vertebrate hosts including small mammals (such as rodents), mammals (e.g. red foxes) and ground-dwelling birds [94]. Bank voles are the most significant hosts for the immature stages of these ticks [147, 210]. Grzybek and colleagues studied TBEV seroprevalence in bank voles using a long-term approach [49]. The most significant factors affecting seroprevalence were the location of vole capture and the year of sampling. Seroprevalence increased notably with increasing host age, with significant interactions observed between these three factors. There was no difference in seroprevalence between the sexes. Based on seroprevalence, the dynamics of TBEV infection vary over time, between local sub-populations of voles, and with host age.

### Zoonotic nematodes

Bank voles also play a role in the circulation of zoonotic nematodes, including *Toxocara* spp. and *Trichinella spiralis*. Biomonitoring focusing on *Toxocara* spp. has revealed the presence and circulation of *Toxocara* spp. agents among bank voles [4]. Older bank voles were more frequently infected than younger individuals, likely due to their longer exposure to environmental contamination with *Toxocara* eggs [73, 114].

Grzybek et al. studied the seroprevalence of *Trichinella* spp. in bank voles from NE Poland, and found a seroprevalence of 1.52% [52]. Seroprevalence was primarily concentrated in one of three study sites and limited to the oldest individuals in the study, but did not differ between the sexes. Although a local prevalence of 1.52% may seem low, when extrapolated to the national population of bank voles during peak years, potentially numbering in the hundreds of millions of animals, the number of infected bank voles is likely to exceed tens of millions on a nationwide scale. Other studies carried out in Europe indicate that bank voles play a minor role in spreading *Trichinella* spp. [216].

The relationship between disease ecology and biological invasions is complex, as invasive species can impact host–parasite dynamics through processes such as parasite spillover to native hosts, parasite spillback from natives back to invaders, and enemy release-concepts emphasized in the review by [232]. This integrated perspective can help predict ecological outcomes, including novel host–parasite interactions, shifts in disease prevalence among communities, and changes in ecosystem health and biodiversity. Research on bioinvasion and parasite transmission continues to examine both invasive and native host species, especially regarding potential transmission of virulent pathogens and their ecological consequences [127, 128, 142, 178].

The findings of Stuart et al. [205] illustrate how the examination of invasive and native hosts, along with the identification of their parasitic communities, allows us to uncover the dynamic processes that influence the parasitic component within a community. The dilution effect, a concept describing how increased biodiversity or changes in host community structure can reduce disease transmission, is well described in general terms by [96]. In the case of bank voles, field studies in Ireland and the UK have shown that their increasing density as an invasive species was associated with reduced parasite burdens, including lower prevalence, richness, and intensity of gastrointestinal helminths and ectoparasites [127, 128, 205]. This may reflect both a dilution effect within multi-host systems and aspects of enemy release, whereby invasive species experience reduced parasitic pressure because they leave behind many of their natural enemies during the invasion process [30]. This phenomenon can contribute to the competitive success of invasive species in novel environments by reducing their parasite-mediated constraints [30].

Moreover, in multispecies small mammal communities (comprising both voles and shrews), it was found that the density of other species reduced the seroprevalence of PUUV in bank voles. However, this effect was observed only in spring to early summer, when populations primarily consisted of overwintered, breeding (and more or less territorial) individuals. In autumn, when most animals were nonbreeding subadults, no dilution effect was detected. This highlights the importance of understanding how varying population structures influence disease ecology [70, 221]. Several crucial factors must not be disregarded in research, including seasonality, which affects the presence of juveniles, hormonal changes, and immunoregulation [204, 205].

Together, these findings underscore the complexity of eco-epidemiological dynamics in bank vole populations, where host community composition, population structure, seasonal fluctuations, and patterns of co-infection influence pathogen transmission. Understanding these interacting factors is essential for predicting zoonotic risk and designing effective disease surveillance strategies in natural systems.

## Eco-evolutionary field experiments in bank voles

The choice of the bank vole as a model organism for eco-evolutionary field experiments is well-justified due to several key factors. It displays significant phenotypic and genetic variation in its life history and physiological characteristics and behaviours [132, 134], making it an ideal candidate for studying selection mechanisms, that can maintain genetic variation and drive evolution in mammalian populations. Secondly, the bank vole is well-suited for breeding under laboratory conditions, which is crucial for experimental evolution studies [182]. In addition to laboratory experiments, field experiments can be conducted using large outdoor enclosures [135]. These enclosures provide a bridge between controlled laboratory settings and natural ecological conditions. By studying bank voles under semi-natural environments, researchers can gain a more comprehensive understanding of their biology, behaviour, and responses to ecological selection mechanisms, for example, the influence of density-dependent processes on phenotypic and genotypic variation [67].

In evolutionary studies, it is crucial to measure the fitness-related traits of individuals (e.g. number and size of offspring) to understand how certain traits or behaviours contribute to their reproductive success and survival. While birds have traditionally been the dominant models in evolutionary studies due to their ease of observation and manipulation [34], it has been demonstrated that such experiments can also be conducted in mammals, particularly in bank voles. By capturing pregnant female bank voles from their natural or seminatural environments and relocating them to laboratory settings, researchers can measure the number and size of offspring produced by each female [135]. After birth, marked mothers and their offspring are returned to their territories. The mother vole is allowed to carry her pups to a new nest. After about three weeks, researchers can return to recapture the mother and her young. This helps assess the female's fitness to her reproductive effort and other factors. The procedure also allows for investigating evolutionary trade-offs, such as those between offspring number and size or a female's reproductive effort and future performance. Researchers can manipulate these trade-offs by altering reproductive effort through hormonal manipulations of gonadotropin hormones before copulation [159] or adjusting litter size after birth [105], The latter can be done by swapping pups between mothers since female bank voles do not seem to recognize their offspring [135]. These manipulations help to assess the effects of altered reproductive effort on female bank voles' fitness and performance under various conditions.

The high trappability of bank voles, often resulting in capture rates of up to 70% within a few trapping nights, offers a valuable opportunity to estimate the fitness of male bank voles [230]. Researchers can utilize this advantage by releasing males into a population comprised of females and subsequently recapturing the females approximately 16–17 days after possible copulations, just before the females give birth [148]. Through paternal analyses of the offspring, researchers can estimate the mating and breeding success of the males and examine their mating behaviour, including instances of multiple mating. This approach also enables the assessment of correlated traits in bank voles, e.g. the trade-off between testosterone levels and immunocompetence [149], providing insights into male strategies in bank vole populations.

Outdoor enclosures play a crucial role in conducting experiments with bank voles [135]. These enclosures provide a controlled environment where researchers can manipulate various factors such as vole densities and frequencies of different tactics within the populations [133]. They also help control the movement of animals, allowing for more reliable estimates of their survival and life-time fitness. To meet the requirements for the bank vole experiments, the outdoor enclosures should be large enough, with a minimum size of 2000 m^2^ each. This size ensures that 4–6 female voles can have enough space for their territories within each enclosure, since having sufficient space is important for the natural behaviour and territorial dynamics of female bank voles [22]. Additionally, multiple enclosures are typically used to provide replication and enhance statistical power (e.g., 4–12 replicates depending on logistical constraints).

When testing evolutionary hypotheses, it is important to measure the relative fitness of individuals, which involves assessing an individual's fitness in relation to its neighbours [163]. The most effective way to assess relative fitness is through experimental designs that generate the conditions for negative frequency-dependent selection under captive conditions. In this design, the frequencies of individuals with two different tactics are manipulated, such that both tactics are either rare or common in the population [95]. If for example, the rare tactic can successfully invade the population, it provides clear evidence that individuals with different tactics affect each other, and in this case, the rare tactic has higher relative fitness caused by some fitness advantages over the common one. Negative frequency-dependent selection has been employed in multiple enclosure experiments involving bank voles [132, 133]. Here we present two examples of studies where this experimental approach has been utilised.

*Infanticidal behaviour in bank voles.* The killing of offspring by conspecifics is observed in various mammalian species and can be attributed to both males and females. However, it is notable that infanticide is particularly common among female bank voles [170]. This behaviour exhibits significant variation between female bank voles in nature, and there is evidence to suggest that it has a high heritability [132]. As a result, researchers can utilize the different genetic behavioural tactics displayed by female bank voles (infanticidal and non-infanticidal) to investigate the potential ecological and evolutionary mechanisms that maintain this variation. According to the resource competition hypothesis, individuals that engage in infanticide or their relatives may gain increased access to resources such as food or nesting sites by eliminating the offspring of competing breeders [212]. To test this hypothesis, an experimental design involving negative frequency-dependent selection on infanticidal tactics in environments with varying levels of food resources was applied [132]. Infanticidal strategy can successfully infiltrate a population of non-infanticidal individuals, but only under conditions of limited food resources. The frequencies of the infanticidal tactic may fluctuate in response to spatial and/or temporal variations in food resources, as well as density variations within bank vole populations. Furthermore, infanticidal behaviour can be an important phenomenon driving synchronous breeding in bank vole populations [170].

*Conflicts between sexes and sexually antagonistic selection*. In bank voles, the selection of males with high behavioural dominance leads to reduced fertility in their sisters (referred to as tactic 1). Conversely, choosing females with high fecundity results in their brothers exhibiting lower dominance when competing with other males (referred to as tactic 2) [150]. To investigate the potential for sexually antagonistic selection, Mokkonen et al. conducted a field experiment using a negative frequency-dependent selection design [152], introducing tactics that were either rare or common within the population. High-dominance males (tactic 1) achieved the highest reproductive success, measured by the number of offspring sired, but only when they were rare within the population. Males, in general, showed negative frequency dependence, while female success was primarily driven by fertility rather than frequency. An effect that would have remained unnoticed under laboratory conditions was unveiled by implementing semi-natural conditions alongside frequency considerations. The data suggest that when dominant males become too common, they face selection pressures that hinder their reproductive success within the population. However, females consistently experience selection pressures aimed at maximizing their fertility. This gives rise to a conflict between males and females when dominant males are favoured, leading to selection against related females. However, this conflict is minimized or even eliminated when selection shifts, favouring subordinate males, which also benefits related females.

Numerous studies have now indicated that the bank vole is an excellent model species for experimentally testing behavioural, physiological and life-history traits involved in alternative strategies (Table 1).

**Table 1:** A future goal is to test the hypothesised life-history and behavioural traits involved in the two alternative strategies in bank voles, as well as the potential selection mechanisms that maintain these strategies in nature

Strategy I	Strategy II	Selection mechanisms
Low reproductive effort (females) Low fecundity / High survival (females) Monogamy (females) High immunocompetence (females) Low basal metabolic rate (females) Inferior in female-female competition High testosterone level/ Low immunocompetence (males) High fecundity / Low survival (males) Superior in male-male competition Higher polygamy (males)	High reproductive effort (females) High fecundity / Low survival (females) Polygamy (females) Low immunocompetence (females) High basal metabolic rate (females) Superior in female-female competition Low testosterone level/ High immunocompetence (males) Low fecundity/High survival (males) Inferior in male-male competition Lower polygamy (Pair-bonding?)(males)	Negative frequency-dependent selection Density-dependent selection Sexually antagonistic selection Sex and strategy specific fecundity versus survival selection

## Conclusions

The bank vole has become an important model organism due to its broad ecological distribution, well-characterized life history, and relevance across multiple biological disciplines. Its well-documented biology—including reproductive ecology, physiology, and behavioural traits—provides a robust foundation for both field-based and experimental research.

Practical protocols for trapping, housing, and breeding bank voles under laboratory conditions have enabled controlled studies while retaining connections to natural systems. These methods support the integration of experimental precision with ecological realism—an important strength of the model.

Recent advances in genetic and genomic resources, including high-quality genome assemblies and transcriptomes, have opened new avenues for research into adaptation, gene function, and evolutionary processes. These tools enable detailed investigations of intraspecific genetic variation, population structure, and the genomic basis of adaptation to environmental change—topics central to understanding and predicting biodiversity responses to global challenges.

The bank vole has also proven valuable in experimental evolution research. Studies of traits such as metabolic efficiency, dietary specialization, and behaviour illustrate how this species can reveal the dynamics of natural selection in both laboratory and semi-natural conditions.

In evolutionary immunogenetics and disease ecology, the bank vole is a powerful system for studying natural variation in immune genes, particularly the MHC, and its relationship to pathogen exposure. Its role as a natural reservoir for zoonotic pathogens such as *Borrelia* spp. and Puumala orthohantavirus further enhances its relevance for understanding host–pathogen coevolution under realistic ecological conditions.

Finally, long-term eco-evolutionary field experiments using bank voles have advanced our understanding of how ecological and evolutionary processes interact. These studies allow for the examination of host density effects, disease dynamics, and selective pressures in real time, providing a framework for testing life-history theory in wild populations.

Collectively, these diverse applications highlight the bank vole’s growing role as a cross-disciplinary model system. Its unique combination of ecological relevance, experimental flexibility, and expanding genomic resources makes it exceptionally well-suited for integrative research. By enabling detailed study of genetic variation, immune dynamics, and life-history traits in both laboratory and natural environments, the bank vole facilitates investigations into how organisms respond to environmental stressors such as climate change, habitat fragmentation, and pathogen pressure. This ability to combine ecological realism with experimental precision ensures the continued value of the bank vole as a model for addressing both fundamental and applied questions in biology.

## Future directions

The bank vole offers exceptional potential for future research across evolutionary biology, ecology, immunology, and disease dynamics. As genomic and experimental resources continue to expand, new opportunities are emerging to address complex, interdisciplinary questions using this versatile model system.

One key direction involves leveraging genomic tools to explore local adaptation and evolutionary responses to environmental change. High-resolution comparative genomics, functional annotation of complex gene families (e.g., immune and metabolic genes), and regulatory elements will deepen our understanding of adaptive mechanisms. Population-scale resequencing across the bank vole’s broad geographic range can reveal how genetic variation is structured along environmental gradients and shaped by selective pressures such as climate, habitat fragmentation, and pathogen diversity.

Integrating these genomic data with ecological, behavioural, and experimental approaches will be critical. For example, combining spatial ecology with genotype–phenotype association studies could help elucidate how organisms adapt to variable environments in real time. Experimental evolution in semi-natural settings, already pioneered using the bank vole, can be expanded with transcriptomic and epigenomic profiling to capture dynamic responses to environmental stress.

The bank vole also holds particular promise for advancing eco-immunology and host–pathogen coevolution. Future work could examine how genetic variation in immune genes, especially the MHC, interacts with infection dynamics across landscapes and seasons. Longitudinal monitoring and experimental infection models may help disentangle causal relationships between immune variation, pathogen load, and fitness in natural populations.

Finally, comparative studies with other small mammal models—such as *Mus musculus*, *Microtus* spp., and *Sorex* spp.–can help contextualize findings from the bank vole and facilitate the transfer of methodological frameworks. Such cross-species approaches will enhance the relevance of bank vole research and extend its utility across a broader spectrum of biological inquiry.

In sum, the bank vole is ideally suited for integrative, question-driven research at the intersection of genomics, ecology, and evolution. By harnessing multidisciplinary methods and scaling up genomic sampling, researchers can fully unlock the potential of this model organism to address both fundamental and applied challenges in biology.

## Data Availability

All data are available within the manuscript.

## References

[1] Abramson NI, Rodchenkova EN, Kostygov AYu. Genetic variation and phylogeography of the bank vole (*Clethrionomys glareolus*, Arvicolinae, Rodentia) in Russia with special reference to the introgression of the mtDNA of a closely related species, red-backed vole (*Cl. rutilus*). Russ J Genet. 2009;45:533–45. 19534420

[2] Abzhanov A, Extavour CG, Groover A, Hodges SA, Hoekstra HE, Kramer EM, Monteiro A. Are we there yet? Tracking the development of new model systems. Trends Genet. 2008;24:353–60. 18514356 10.1016/j.tig.2008.04.002

[3] Al-Qazaz D. Flea community and their role as vectors of Bartonella bacteria in three isolated populations of bank voles from the Masuria Lake District. BSc Thesis, Warsaw University, Warsaw, 2020.

[4] Antolová D, Reiterová K, Stanko M, Zalesny G, Fričová J, Dvorožňáková E. Small mammals: paratenic hosts for species of Toxocara in Eastern Slovakia. J Helminthol. 2013;87:52–8. 22284742 10.1017/S0022149X11000848

[5] Arslan A, Zima J. Karyotypes of the mammals of Turkey and neighbouring regions: a review. Folia Zool. 2014;63:1–62.

[6] Axtner J, Sommer S. Gene duplication, allelic diversity, selection processes and adaptive value of MHC class II DRB genes of the bank vole, *Clethrionomys glareolus*. Immunogenetics. 2007;59:417–26. 17351770 10.1007/s00251-007-0205-y

[7] Babik W, Stuglik M, Qi W, Kuenzli M, Kuduk K, Koteja P, Radwan J. Heart transcriptome of the bank vole (*Myodes glareolus*): towards understanding the evolutionary variation in metabolic rate. BMC Genomics. 2010;11:390. 20565972 10.1186/1471-2164-11-390 PMC2996923

[8] Babik W, Taberlet P, Ejsmond MJ, Radwan J. New generation sequencers as a tool for genotyping of highly polymorphic multilocus MHC system. Mol Ecol Resour. 2009;9:713–9. 21564729 10.1111/j.1755-0998.2009.02622.x

[9] Bajer A. Between-year variation and spatial dynamics of Cryptosporidium spp. and Giardia spp. infections in naturally infected rodent populations. Parasitology. 2008;135:1629–49. 18992178 10.1017/S0031182008004952

[10] Bajer A, Bednarska M, Pawełczyk A, Behnke JM, Gilbert FS, Sinski E. Prevalence and abundance of *Cryptosporidium parvum* and Giardia spp. in wild rural rodents from the mazury lake district region of Poland. Parasitology. 2002;125:21–34. 12166517 10.1017/s0031182002001865

[11] Bajer A, Behnke JM, Bednarska M, Kuliś K, Siński E. Co-occurrence of *Cryptosporidium parvum*, Giardia spp. and helminth in populations of small rodents. Wiadomości Parazytologiczne. 2004;50:307–15. 16859041

[12] Bajer A, Cacciò S, Bednarska M, Behnke JM, Pieniazek NJ, Sinski E. Preliminary molecular characterization of *Cryptosporidium parvum* isolates of wildlife rodents from Poland. J Parasitol. 2003;89:1053–5. 14627156 10.1645/GE-3096RN

[13] Bajer A, Pawelczyk A, Behnke JM, Gilbert FS, Sinski E. Factors affecting the component community structure of haemoparasites in bank voles (*Clethrionomys glareolus*) from the mazury lake district region of Poland. Parasitology. 2001;122(Pt 1):43–54. 11197763 10.1017/s0031182000007058

[14] Bajer A, Welc-Faleciak R, Bednarska M, Alsarraf M, Behnke-Borowczyk J, Siński E, Behnke JM. Long-term spatiotemporal stability and dynamic changes in the haemoparasite community of bank voles (*Myodes glareolus*) in NE Poland. Microb Ecol. 2014;68:196–211. 24604428 10.1007/s00248-014-0390-9 PMC4103999

[15] Behnke JM, Bajer A, Harris PD, Newington L, Pidgeon E, Rowlands G, Sheriff C, Kuliś-Malkowska K, Siński E, Gilbert FS, Barnard CJ. Temporal and between-site variation in helminth communities of bank voles (*Myodes glareolus*) from N.E. Poland. 1. Regional fauna and component community levels. Parasitology. 2008;135:985–97. 18598578 10.1017/S0031182008004393

[16] Behnke JM, Barnard CJ, Bajer A, Bray D, Dinmore J, Frake K, Osmond J, Race T, Sinski E. Variation in the helminth community structure in bank voles (*Clethrionomys glareolus*) from three comparable localities in the mazury lake istrict region of Poland. Parasitology. 2001;123:401–14. 11676372 10.1017/s0031182001008605

[17] Boratyński Z, Alves P, Berto S, Koskela E, Mappes T, Melo-Ferreira J. Introgression of mitochondrial DNA among Myodes voles: consequences for energetics? BMC Evol Biol. 2011;11:355. 22151479 10.1186/1471-2148-11-355 PMC3260118

[18] Bordenstein SR, Network THB, Gilbert MTP, Ginnan N, Malacrinò A, Martino ME, Bahrndorff S, Mundra S, Martin MD, Theis KR, Hird SM, Caro-Quintero A, Sharpton TJ, Kohl KD, Barnes CJ, et al. The disciplinary matrix of holobiont biology. Science. 2024;386:731–2. 39541453 10.1126/science.ado2152

[19] Bourret V, Dutra L, Alburkat H, Mäki S, Lintunen E, Wasniewski M, Kant R, Grzybek M, Venkat V, Asad H, Pradel J, Bouilloud M, Leirs H, Colombo VC, Sluydts V, et al. Serologic surveillance for SARS-CoV-2 infection among wild rodents. Euro Emerg Infect Dis. 2022;28(12):2577. 10.3201/eid2812.221235 PMC9707589 36322954

[20] Bryda EC. The mighty mouse: the impact of rodents on advances in biomedical research. Mo Med. 2013;110:207–11. 23829104 PMC3987984

[21] Buchalczyk A. Reproduction, mortality and longevity of the bank vole under laboratory conditions. Acta Theriol. 1970;15:1–12.

[22] Bujalska G, Grüm L. Social organization of the bank vole (*Clethrionomys glareolus*, Schreber 1780) and its demographic consequences: a model. Oecologia. 1989;80(1):70–81. 23494348 10.1007/BF00789934

[23] Bujalska G, Saitoh T. Territoriality and its consequences. Pol J Ecol. 2000;48:37–49.

[24] Butet A, Delettre YR. Diet differentiation between European arvicoline and murine rodents. Acta Theriol. 2011;56:297.

[25] Calamari ZT, Song A, Cohen E, Akter M, Das Roy R, Hallikas O, Christensen MM, Li P, Marangoni P, Jernvall J, Klein OD. Bank vole genomics links determinate and indeterminate growth of teeth. BMC Genomics. 2024;25:1000. 39472825 10.1186/s12864-024-10901-2 PMC11523675

[26] Cayol C, Giermek A, Gomez-Chamorro A, Hytönen J, Kallio ER, Mappes T, Salo J, Voordouw MJ, Koskela E. *Borrelia afzelii* alters reproductive success in a rodent host. Proc R Soc B Biological Sci. 2018;285:20181056. 10.1098/rspb.2018.1056 PMC6111163 30068677

[27] Chantrey J, Meyer H, Baxby D, Begon M, Bown KJ, Hazel SM, Jones T, Montgomery WI, Bennett M. Cowpox: reservoir hosts and geographic range. Epidemiol Infect. 1999;122:455–60. 10459650 10.1017/s0950268899002423 PMC2809641

[28] Chrząścik KM, Sadowska ET, Rudolf A, Koteja P. Learning ability in bank voles selected for high aerobic metabolism, predatory behaviour and herbivorous capability. Physiol Behav. 2014;135:143–51. 24952259 10.1016/j.physbeh.2014.06.007

[29] Čížková D, de Bellocq JG, Baird SJE, Piálek J, Bryja J. Genetic structure and contrasting selection pattern at two major histocompatibility complex genes in wild house mouse populations. Heredity. 2011;106:727–40. 20823902 10.1038/hdy.2010.112 PMC3186229

[30] Colautti RI, Ricciardi A, Grigorovich IA, MacIsaac HJ. Is invasion success explained by the enemy release hypothesis? Ecol Lett. 2004;7:721–33.

[31] Cornetti L, Tschirren B. Combining genome-wide association study and F ST-based approaches to identify targets of Borrelia -mediated selection in natural rodent hosts. Mol Ecol. 2020;29:1386–97. 32163646 10.1111/mec.15410

[32] Cornulier T, Yoccoz NG, Bretagnolle V, Brommer JE, Butet A, Ecke F, Elston DA, Framstad E, Henttonen H, Hörnfeldt B, Huitu O, Imholt C, Ims RA, Jacob J, Jędrzejewska B, et al. Europe-wide dampening of population cycles in keystone herbivores. Science. 2013;340:63–6. 23559246 10.1126/science.1228992

[33] Dahmana H, Granjon L, Diagne C, Davoust B, Fenollar F, Mediannikov O. Rodents as hosts of pathogens and related zoonotic disease risk. Pathogens. 2020;9:202. 32164206 10.3390/pathogens9030202 PMC7157691

[34] Davies NB, Krebs JR, West SA. An introduction to behavioural ecology. 4th ed. Berlin: Wiley; 2013.

[35] Dheyongera G, Grzebyk K, Rudolf AM, Sadowska ET, Koteja P. The effect of chlorpyrifos on thermogenic capacity of bank voles selected for increased aerobic exercise metabolism. Chemosphere. 2016;149:383–90. 26878110 10.1016/j.chemosphere.2015.12.120

[36] Eccard JA, Ylönen H. Initiation of breeding after winter in bank voles: effects of food and population density. Can J Zool. 2001;79:1743–53.

[37] Escalante MA, Marková S, Searle JB, Kotlík P. Genic distribution modelling predicts adaptation of the bank vole to climate change. Commun Biol. 2022;5:981. 36114276 10.1038/s42003-022-03935-3 PMC9481625

[38] Feore SM, Bennett M, Chantrey J, Jones T, Baxb D, Begon M. The effect of cowpox virus infection on fecundity in bank voles and wood mice. Proc R Soc Lond B. 1997;264:1457–61. 10.1098/rspb.1997.0202 PMC1688698 9364786

[39] Forbes KM, Voutilainen L, Jääskeläinen A, Sironen T, Kinnunen PM, Stuart P, Vapalahti O, Henttonen H, Huitu O. Serological survey of rodent-borne viruses in Finnish field voles. Vector Borne Zoonotic Dis. 2014;14:278–83. 24689532 10.1089/vbz.2013.1526 PMC3993079

[40] Franke A, Ulrich RG, Weber S, Osterrieder N, Keller M, Hoffmann D, Beer M. Experimental cowpox virus (CPXV) infections of bank voles: exceptional clinical resistance and variable reservoir competence. Viruses. 2017;9:391. 29257111 10.3390/v9120391 PMC5744165

[41] Gallo G, Kotlik P, Roingeard P, Monot M, Chevreux G, Ulrich RG, Tordo N, Ermonval M. Diverse susceptibilities and responses of human and rodent cells to orthohantavirus infection reveal different levels of cellular restriction. PLoS Negl Trop Dis. 2022;16: e0010844. 36223391 10.1371/journal.pntd.0010844 PMC9591050

[42] Gębczyńska Z. Food habits of the bank vole and phenological phases of plants in an oak-hornbeam forest. Acta Theriol. 1976;21:223–36.

[43] Gipps JHW. Behavior of bank voles, *Clethrionomys glareolus*, in the field. J Mammal. 1981;62:382–4.

[44] Gliwicz J. Dispersal in bank voles: benefits to emigrants or to residents? Acta Theriol. 1993;38:31–8.

[45] Gomez-Chamorro A, Battilotti F, Cayol C, Mappes T, Koskela E, Boulanger N, Genné D, Sarr A, Voordouw MJ. Susceptibility to infection with Borrelia afzelii and TLR2 polymorphism in a wild reservoir host. Sci Rep. 2019;9:6711. 31040326 10.1038/s41598-019-43160-3 PMC6491475

[46] Gomez-Chamorro A, Heinrich V, Sarr A, Roethlisberger O, Genné D, Bregnard C, Jacquet M, Voordouw MJ. Maternal antibodies provide bank voles with strain–specific protection against infection by the lyme disease pathogen. Appl Environ Microbiol. 2019;85:e01887-e1919. 31540991 10.1128/AEM.01887-19 PMC6856323

[47] Goszczynski J. Diet of foxes and martens in central Poland. Acta Theriol. 1986;31:491–506.

[48] Grosiak M, Koteja P, Bauchinger U, Sadowska ET. Age-related changes in the thermoregulatory properties in bank voles from a selection experiment. Front Physiol. 2020;11:576304. 33329026 10.3389/fphys.2020.576304 PMC7711078

[49] Grzybek M, Alsarraf M, Tołkacz K, Behnke-Borowczyk J, Biernat B, Stańczak J, Strachecka A, Guz L, Szczepaniak K, Paleolog J, Behnke JM, Bajer A. Seroprevalence of TBEV in bank voles from Poland—a long-term approach. Emerg Microb Infect. 2018;7:1–8. 10.1038/s41426-018-0149-3 PMC6092418 30108201

[50] Grzybek M, Antolová D, Tołkacz K, Alsarraf M, Behnke-Borowczyk J, Nowicka J, Paleolog J, Biernat B, Behnke JM, Bajer A. Seroprevalence of *Toxoplasma gondii* among sylvatic rodents in Poland. Animals. 2021;11:1048. 33917803 10.3390/ani11041048 PMC8068096

[51] Grzybek M, Bajer A, Bednarska M, Al-Sarraf M, Behnke-Borowczyk J, Harris PD, Price SJ, Brown GS, Osborne S-J, Siński E, Behnke JM. Long-term spatiotemporal stability and dynamic changes in helminth infracommunities of bank voles (*Myodes glareolus*) in NE Poland. Parasitology. 2015;142:1722–43. 26442655 10.1017/S0031182015001225

[52] Grzybek M, Cybulska A, Tołkacz K, Alsarraf M, Behnke-Borowczyk J, Szczepaniak K, Strachecka A, Paleolog J, Moskwa B, Behnke JM, Bajer A. Seroprevalence of Trichinella spp. infection in bank voles (*Myodes glareolus*)—a long term study. Int J Parasitol Parasites Wildl. 2019;9:144–8. 31193257 10.1016/j.ijppaw.2019.03.005 PMC6524169

[53] Guivier E, Galan M, Henttonen H, Cosson J-F, Charbonnel N. Landscape features and helminth co-infection shape bank vole immunoheterogeneity, with consequences for puumala virus epidemiology. Heredity. 2014;112:274–81. 24149655 10.1038/hdy.2013.103 PMC3931171

[54] Gustafsson T, Andersson B, Meurling P. Effect of social rank on the growth of the preputial glands in male bank voles, *Clethrionomys glareolus*. Physiol Behav. 1980;24:689–92. 6994144 10.1016/0031-9384(80)90398-4

[55] Gustafsson TO, Andersson CB, Westlin LM. Reproduction in laboratory colonies of bank vole, *Clethrionomys glareolus*, originating from populations with different degrees of cyclicity. Oikos. 1983;40:182.

[56] Hall SJG. Haemoglobin polymorphism in the bank vole, *Clethrionomys glareolus*, in Britain. J Zool. 1979;187:153–60.

[57] Hämäläinen R, Kajanus MH, Forsman JT, Kivelä SM, Seppänen J, Loukola OJ. Ecological and evolutionary consequences of selective interspecific information use. Ecol Lett. 2023;26:490–503. 36849224 10.1111/ele.14184

[58] Hampton T. Bank vole a novel model of prion transmission. JAMA. 2014;311:1722.

[59] Hanhimäki E, Watts PC, Koskela E, Koteja P, Mappes T, Hämäläinen AM. Evolved high aerobic capacity has context-specific effects on gut microbiota. Front Ecol Evol. 2022;10:934164.

[60] Hanski I, Hansson L, Henttonen H. Specialist predators, generalist predators, and the microtine rodent cycle. J Anim Ecol. 1991;60:353.

[61] Hanski I, Henttonen H, Hansson L. Temporal variability and geographical patterns in the population density of microtine rodents: a reply to xia and boonstra. Am Nat. 1994;144:329–42.

[62] Hansson L. Breeding of captive bank voles (*Clethrionomys glareolus*) related to dynamics of source populations. Reproduction. 1990;89:769–72. 10.1530/jrf.0.0890769 2205723

[63] Hansson L, Henttonen H. Regional differences in cyclicity and reproduction in *Clethrionomys* species: are they related? Ann Zool Fenn. 1985;22:277–88.

[64] Haukisalmi V, Henttonen H. The impact of climatic factors and host density on the long-term population dynamics of vole helminths. Oecologia. 1990;83:309–15. 28313000 10.1007/BF00317553

[65] Haukisalmi V, Henttonen H. Variability of helminth assemblages and populations in the bank vole *Clethrionomys glareolus*. Pol J Ecol. 2000;48:219–31.

[66] Haukisalmi V, Henttonen H, Tenora F. Population dynamics of common and rare helminths in cyclic vole populations. J Anim Ecol. 1988;57:807.

[67] Helle H, Koskela E, Mappes T. Life in varying environments: experimental evidence for delayed effects of juvenile environment on adult life history. J Anim Ecol. 2012;81:573–82. 22191455 10.1111/j.1365-2656.2011.01937.x

[68] Henderson ND. Spurious associations in unreplicated selected lines. Behav Genet. 1997;27:145–54. 9145553 10.1023/a:1025689425738

[69] Henttonen H. Long-term dynamics of the bank vole *Clethrionomys glareolus* at Pallasjärvi, Northern Finnish taiga. Polish J Ecol. 2000;48:87–96.

[70] Henttonen H. Importance of demography in understanding disease ecology in small mammals. Therya. 2022;13:33–8.

[71] Henttonen H, Oksanen T, Jortikka A, Haukisalmi V. How much do weasels shape microtine cycles in the Northern Fennoscandian taiga? Oikos. 1987;50:353.

[72] Hickman DL, Johnson J, Vemulapalli TH, Crisler JR, Shepherd R. Commonly used animal models. Princ Anim Res. 2017; 117–175. 10.1016/B978-0-12-802151-4.00007-4

[73] Hildebrand J, Zalesny G, Okulewicz A, Baszkiewicz K. Preliminary studies on the zoonotic importance of rodents as a reservoir of toxocariasis from recreation grounds in Wroclaw (Poland). Helminthologia. 2009;46:80–4.

[74] Hörnfeldt B. Long-term decline in numbers of cyclic voles in boreal Sweden: analysis and presentation of hypotheses. Oikos. 2004;107:376–92.

[75] Horníková M, Lanier HC, Marková S, Escalante MA, Searle JB, Kotlík P. Genetic admixture drives climate adaptation in the bank vole. Commun Biol. 2024;7:863. 39009753 10.1038/s42003-024-06549-z PMC11251159

[76] Horníková M, Marková S, Lanier HC, Searle JB, Kotlík P. A dynamic history of admixture from Mediterranean and Carpathian glacial refugia drives genomic diversity in the bank vole. Ecol Evol. 2021;11:8215–25. 34188881 10.1002/ece3.7652 PMC8216894

[77] Hornok S, Daccord J, Takács N, Kontschán J, Tuska-Szalay B, Sándor AD, Szekeres S, Meli ML, Hofmann-Lehmann R. Investigation on haplotypes of ixodid ticks and retrospective finding of Borrelia miyamotoi in bank vole (*Myodes glareolus*) in Switzerland. Ticks Tick Borne Dis. 2022;13: 101865. 34814063 10.1016/j.ttbdis.2021.101865

[78] Hseiky A, Sadowska ET, Koteja P. The effects of short‐term consumption of a Western diet on aerobic exercise performance in bank voles with inherently distinct metabolic rates. Experimental Physiology 2025 DOI: 10.1113/EP092646 10.1113/EP092646 PMC12949117 40714913

[79] Hughes DJ, Kipar A, Leeming G, Sample JT, Stewart JP. Experimental infection of laboratory-bred bank voles (*Myodes glareolus*) with murid herpesvirus 4. Adv Virol. 2012;157:2207–12. 10.1007/s00705-012-1397-5 22782137

[80] Hutterer R, Kryštufek B, Yigit N, Mitsainas G, Palomo L, Henttonen H, Vohralík V, Zagorodnyuk I, Juškaitis R, Meinig H, Bertolino S. *Myodes glareolus* (amended version of 2016 assessment). The IUCN Red List of Threatened Species. 2021. 10.2305/IUCN.UK.2021-1.RLTS.T4973A197520967.en.

[81] Ivanter EV. The reproductive ecology of the bank vole *Myodes (Clethrionomys) glareolus* Schreb. in North periphery of its areal: I. Sex cycles, course, dates, and intensive reproduction. Biol Bull. 2020;47:535–47.

[82] IUCN. The IUCN Red List of Threatened Species. Version 2021-1. 2021. Available at: www.iucnredlist.org.

[83] Jaromin E, Sadowska ET, Koteja P. A dopamine and noradrenaline reuptake inhibitor (bupropion) does not alter exercise performance of bank voles. Curr Zool. 2016;62:307–15. 29491918 10.1093/cz/zow026 PMC5804238

[84] Jaromin E, Sadowska ET, Koteja P. Is experimental evolution of an increased aerobic exercise performance in bank voles mediated by endocannabinoid signaling pathway? Front Physiol. 2019;10:640. 31191344 10.3389/fphys.2019.00640 PMC6546880

[85] Jaromin E, Sadowska ET, Koteja P. The effect of monoamines reuptake inhibitors on aerobic exercise performance in bank voles from a selection experiment. Curr Zool. 2019;65:409–19. 31413714 10.1093/cz/zoy063 PMC6688583

[86] Jaromin E, Wyszkowska J, Labecka AM, Sadowska ET, Koteja P. Hind limb muscle fibre size and glycogen stores in bank voles with increased aerobic exercise metabolism. J Exp Biol. 2015;219(4):470–3. 26685167 10.1242/jeb.130476

[87] Jernfors T, Danforth J, Kesäniemi J, Lavrinienko A, Tukalenko E, Fajkus J, Dvořáčková M, Mappes T, Watts PC. Expansion of rDNA and pericentromere satellite repeats in the genomes of bank voles *Myodes glareolus* exposed to environmental radionuclides. Ecol Evol. 2021;11:8754–67. 34257925 10.1002/ece3.7684 PMC8258220

[88] Jusko M, Flea community and their role as vectors of bacteria Bartonella sp. in three sympatric populations of voles from the Mazury lake district, BSc thesis, Warsaw University, Warsaw, 2015.

[89] Kallio ER, Begon M, Henttonen H, Koskela E, Mappes T, Vaheri A, Vapalahti O. Hantavirus infections in fluctuating host populations: the role of maternal antibodies. Proc R Soc B Biol Sci. 2010;277:3783–91. 10.1098/rspb.2010.1022 PMC2992709 20591866

[90] Kallio ER, Henttonen H, Koskela E, Lundkvist Å, Mappes T, Vapalahti O. Maternal antibodies contribute to sex-based difference in hantavirus transmission dynamics. Biol Let. 2013;9:20130887. 24352416 10.1098/rsbl.2013.0887 PMC3871379

[91] Kallio ER, Klingström J, Gustafsson E, Manni T, Vaheri A, Henttonen H, Vapalahti O, Lundkvist Å. Prolonged survival of Puumala hantavirus outside the host: evidence for indirect transmission via the environment. J Gen Virol. 2006;87:2127–34. 16847107 10.1099/vir.0.81643-0

[92] Kallio ER, Poikonen A, Vaheri A, Vapalahti O, Henttonen H, Koskela E, Mappes T. Maternal antibodies postpone hantavirus infection and enhance individual breeding success. Proc R Soc B Biological Sci. 2006;273:2771–6. 10.1098/rspb.2006.3645 PMC1635497 17015326

[93] Kallio ER, Voutilainen L, Vapalahti O, Vaheri A, Henttonen H, Koskela E, Mappes T. Endemic hantavirus infection impairs the winter survival of its rodent host. Ecology. 2007;88:1911–6. 17824420 10.1890/06-1620.1

[94] Karbowiak G, Biernat B. The role of particular tick developmental stages in the circulation of tick-borne pathogens affecting humans in central Europe. 2 Tick-borne encephalitis virus. Ann Parasitol. 2016;62:3–9. 27262951 10.17420/ap6201.25

[95] Kassen R. The experimental evolution of specialists, generalists, and the maintenance of diversity. J Evol Biol. 2002;15:173–90.

[96] Keesing F, Ostfeld RS. Dilution effects in disease ecology. Ecol Lett. 2021;24:2490–505. 34482609 10.1111/ele.13875 PMC9291114

[97] Khalil H, Ecke F, Evander M, Bucht G, Hörnfeldt B. Population dynamics of bank voles predicts human puumala hantavirus risk. EcoHealth. 2019;16:545–57. 31309365 10.1007/s10393-019-01424-4 PMC6858908

[98] Kiani AK, Pheby D, Henehan G, Brown R, Sieving P, Sykora P, Marks R, Falsini B, Capodicasa N, Miertus S, Lorusso L, Dondossola D, Tartaglia GM, Ergoren MC, Dundar M, et al. Ethical considerations regarding animal experimentation. J Prev Med Hyg. 2022;63:E255–66. 36479489 10.15167/2421-4248/jpmh2022.63.2S3.2768 PMC9710398

[99] Kinnunen PM, Henttonen H, Hoffmann B, Kallio ER, Korthase C, Laakkonen J, Niemimaa J, Palva A, Schlegel M, Ali HS, Suominen P, Ulrich RG, Vaheri A, Vapalahti O. Orthopox virus infections in Eurasian wild rodents. Vector Borne Zoonotic Dis. 2011;11(8):1133–40. 21453121 10.1089/vbz.2010.0170

[100] Kivistö R, Kämäräinen S, Huitu O, Niemimaa J, Henttonen H. Zoonotic Cryptosporidium spp in wild rodents and shrews. Microorganisms. 2021;9:2242. 34835368 10.3390/microorganisms9112242 PMC8618411

[101] Klimpel S, Förster M, Schmahl G. Parasites of two abundant sympatric rodent species in relation to host phylogeny and ecology. Parasitol Res. 2007;100:867–75. 17120043 10.1007/s00436-006-0368-8

[102] Klimpel S, Förster M, Schmahl G. Parasite fauna of the bank vole (*Clethrionomys glareolus*) in an urban region of Germany: reservoir host of zoonotic metazoan parasites? Parasitol Res. 2007;102:69–75. 17849150 10.1007/s00436-007-0725-2

[103] Kohl KD, Sadowska ET, Rudolf AM, Dearing MD, Koteja P. Experimental evolution on a wild mammal species results in modifications of gut microbial communities. Front Microbiol. 2016;7:634. 27199960 10.3389/fmicb.2016.00634 PMC4854874

[104] Koivisto E, Huitu O, Korpimäki E. Smaller microtus vole species competitively superior in the absence of predators. Oikos. 2007;116:156–62.

[105] Koivula M, Koskela E, Mappes T, Oksanen TA. Cost of reproduction in the wild: manipulation of reproductive effort in the bank vole. Ecology. 2003;84:398–405.

[106] Konczal M, Babik W, Radwan J, Sadowska ET, Koteja P. Initial molecular-level response to artificial selection for increased aerobic metabolism occurs primarily through changes in gene expression. Mol Biol Evol. 2015;32:1461–73. 25739734 10.1093/molbev/msv038

[107] Konczal M, Koteja P, Orlowska-Feuer P, Radwan J, Sadowska ET, Babik W. Genomic response to selection for predatory behavior in a mammalian model of adaptive radiation. Mol Biol Evol. 2016;33:2429–40. 27401229 10.1093/molbev/msw121

[108] Korpela K, Helle P, Henttonen H, Korpimäki E, Koskela E, Ovaskainen O, Pietiäinen H, Sundell J, Valkama J, Huitu O. Predator–vole interactions in northern Europe: The role of small mustelids revised. Proc R Soc B Biol Sci. 2014;281:20142119. 10.1098/rspb.2014.2119 PMC4241000 25355481

[109] Koskela E, Mappes T, Ylonen H. Territorial behaviour and reproductive success of bank vole *Clethrionomys glareolus* females. J Anim Ecol. 1997;66:341–9.

[110] Kotlík P, Deffontaine V, Mascheretti S, Zima J, Michaux JR, Searle JB. A Northern glacial refugium for bank voles (*Clethrionomys glareolus*). Proc Natl Acad Sci USA. 2006;103:14860–4. 17001012 10.1073/pnas.0603237103 PMC1595441

[111] Kotlík P, Marková S, Horníková M, Escalante MA, Searle JB. The bank vole (*Clethrionomys glareolus*) as a model system for adaptive phylogeography in the european theater. Front Ecol Evol. 2022;10:866605.

[112] Kotlík P, Marková S, Konczal M, Babik W, Searle JB. Genomics of end-Pleistocene population replacement in a small mammal. Proc R Soc B Biol Sci. 2018;285:20172624. 10.1098/rspb.2017.2624 PMC5829201 29436497

[113] Kotlík P, Marková S, Vojtek L, Stratil A, Šlechta V, Hyršl P, Searle JB. Adaptive phylogeography: functional divergence between haemoglobins derived from different glacial refugia in the bank vole. Proc R Soc B Biol Sci. 2014;281:20140021. 10.1098/rspb.2014.0021 PMC4046400 24827438

[114] Krupińska M, Antolová D, Tołkacz K, Szczepaniak K, Strachecka A, Goll A, Nowicka J, Baranowicz K, Bajer A, Behnke JM, Grzybek M. Grassland versus forest dwelling rodents as indicators of environmental contamination with the zoonotic nematode Toxocara spp. Sci Rep. 2023;13:483. 36627309 10.1038/s41598-022-23891-6 PMC9832041

[115] Kryštufek B, Shenbrot G. Voles and lemmings (arvicolinae) of the palaearctic region. Maribor: University of Maribor; 2022. p. 449.

[116] Kryštufek B, Tesakov AS, Lebedev VS, Bannikova AA, Abramson NI, Shenbrot G. Back to the future: the proper name for red-backed voles is *Clethrionomys tilesius* and not *Myodes pallas*. Mammalia. 2020;84:214–7.

[117] Larsen GD. Bank voles accrue scientific interest. Lab Anim. 2016;45:285–285. 10.1038/laban.1070 27439091

[118] Lindquist L, Vapalahti O. Tick-borne encephalitis. Lancet. 2008;371:1861–71. 18514730 10.1016/S0140-6736(08)60800-4

[119] Ling J, Lundeberg EE, Wasberg A, Faria IR, Vucicevic S, Settergren B, Lundkvist Å. Nephropathia epidemica caused by puumala virus in bank voles, Scania. South Swed Emerg Infect Dis. 2024;30(4):732. 10.3201/eid3004.231414 PMC10977816 38526134

[120] Linnenbrink M, Teschke M, Montero I, Vallier M, Tautz D. Meta-populational demes constitute a reservoir for large MHC allele diversity in wild house mice (*Mus musculus*). Front Zool. 2018;15:15. 29721030 10.1186/s12983-018-0266-9 PMC5910556

[121] Lipowska MM, Dheyongera G, Sadowska ET, Koteja P. Experimental evolution of aerobic exercise performance and hematological traits in bank voles. Comp Biochem Physiol A Mol Integr Physiol. 2019;234:1–9. 31004810 10.1016/j.cbpa.2019.04.008

[122] Lipowska MM, Sadowska ET, Bauchinger U, Goymann W, Bober-Sowa B, Koteja P. Does selection for behavioral and physiological performance traits alter glucocorticoid responsiveness in bank voles? J Exp Biol. 2020;223(15):jeb19865. 10.1242/jeb.219865 32561625

[123] Lipowska MM, Sadowska ET, Kohl KD, Koteja P. Experimental evolution of a mammalian holobiont? genetic and maternal effects on the cecal microbiome in bank voles selectively bred for herbivorous capability. Ecol Evolut Physiol. 2024;97:274–91. 10.1086/732781 39680902

[124] Lipowska MM, Sadowska ET, Palme R, Koteja P. Evolution of an increased performance under acute challenge does not exacerbate vulnerability to chronic stress. Sci Rep. 2022;12:2126. 35136150 10.1038/s41598-022-06060-7 PMC8825808

[125] Llobet T, Velikov I, Sogorb L, Peacock F, Jutglar F, Mascarell A, Martí B, Rodríguez-Osorio J. All the Mammals of the World. LYNX NATURE BOOKS; 2023.

[126] Loxley GM, Unsworth J, Turton MJ, Jebb A, Lilley KS, Simpson DM, Rigden DJ, Hurst JL, Beynon RJ. Glareosin: a novel sexually dimorphic urinary lipocalin in the bank vole *Myodes glareolus*. Open Biol. 2017;7(9):170135. 28878040 10.1098/rsob.170135 PMC5627053

[127] Loxton KC, Lawton C, Stafford P, Holland CV. Reduced helminth parasitism in the introduced bank vole (*Myodes glareolus*): more parasites lost than gained. Int J Parasitol Parasites Wildl. 2016;5:175–83. 27408800 10.1016/j.ijppaw.2016.05.005 PMC4925433

[128] Loxton KC, Lawton C, Stafford P, Holland CV. Parasite dynamics in an invaded ecosystem: helminth communities of native wood mice are impacted by the invasive bank vole. Parasitology. 2017;144:1476–89. 28653589 10.1017/S0031182017000981

[129] Maaz D, Krücken J, Blümke J, Richter D, McKay-Demeler J, Matuschka F-R, Hartmann S, von Samson-Himmelstjerna G. Factors associated with diversity, quantity and zoonotic potential of ectoparasites on urban mice and voles. PLoS ONE. 2018;13: e0199385. 29940047 10.1371/journal.pone.0199385 PMC6016914

[130] Macholán M, Baird S, Munclinger P, Piálek J. Evolution of the house mouse. In: Cambridge studies in morphology and molecules: new paradigms in evolutionary biology. Cambridge University Press; 2012. p. 3. 10.1017/CBO9781139044547.

[131] Maiti U, Sadowska ET, ChrzĄścik KM, Koteja P. Experimental evolution of personality traits: open-field exploration in bank voles from a multidirectional selection experiment. Curr Zool. 2019;65:375–84. 31413710 10.1093/cz/zoy068 PMC6688576

[132] Mappes T, Aspi J, Koskela E, Mills SC, Poikonen T, Tuomi J. Advantage of rare infanticide strategies in an invasion experiment of behavioural polymorphism. Nat Commun. 2012;3:611. 22215086 10.1038/ncomms1613 PMC3272565

[133] Mappes T, Koivula M, Koskela E, Oksanen TA, Savolainen T, Sinervo B. Frequency and density-dependent selection on life-history strategies—a field experiment. PLoS ONE. 2008;3: e1687. 18301764 10.1371/journal.pone.0001687 PMC2246017

[134] Mappes T, Koskela E. genetic basis of the trade-off between offspring number and quality in the bank vole. Evolution. 2004;58:645–50. 15119447

[135] Mappes T, Koskela E, Ylönen H. Reproductive costs and litter size in the bank vole. Proc R Soc Lond B. 1995;261:19–24. 10.1098/rspb.1995.0111 7644546

[136] Marchlewska-Koj A, Kolodziej B, Filimowska A. Aggressive behavior of adult bank voles (*Clethrionomys glareolus*) towards conspecifics. Aggressive Behav. 1989;15:381–7.

[137] Marchlewska-Koj A, Kruczek M, Olejniczak P. Mating behaviour of bank voles (*Clethrionomys glareolus*) modified by hormonal and social factors. Mamm Biol. 2003;68:144–52.

[138] Marková S, Horníková M, Lanier HC, Henttonen H, Searle JB, Weider LJ, Kotlík P. High genomic diversity in the bank vole at the northern apex of a range expansion: the role of multiple colonizations and end-glacial refugia. Mol Ecol. 2020;29:1730–44. 32248595 10.1111/mec.15427

[139] Marková S, Lanier HC, Escalante MA, da Cruz MOR, Horníková M, Konczal M, Weider LJ, Searle JB, Kotlík P. Local adaptation and future climate vulnerability in a wild rodent. Nat Commun. 2023;14:7840. 38030627 10.1038/s41467-023-43383-z PMC10686993

[140] Marková S, Searle JB, Kotlík P. Relaxed functional constraints on triplicate α-globin gene in the bank vole suggest a different evolutionary history from other rodents. Heredity. 2014;113:64–73. 24595364 10.1038/hdy.2014.12 PMC4815648

[141] Martinoli A, Preatoni DG, Chiarenzi B, Wauters LA, Tosi G. Diet of stoats (*Mustela erminea*) in an alpine habitat: the importance of fruit consumption in summer. Acta Oecol. 2001;22:45–53.

[142] McManus A, Holland CV, Henttonen H, Stuart P. The invasive bank vole (*Myodes glareolus*): a model system for studying parasites and ecoimmunology during a biological invasion. Animals. 2021;11:2529. 34573495 10.3390/ani11092529 PMC8464959

[143] Meyer BJ, Schmaljohn CS. Persistent hantavirus infections: characteristics and mechanisms. Trends Microbiol. 2000;8:61–7. 10664598 10.1016/s0966-842x(99)01658-3

[144] Migalska M, Sebastian A, Konczal M, Kotlík P, Radwan J. De novo transcriptome assembly facilitates characterisation of fast-evolving gene families, MHC class I in the bank vole (*Myodes glareolus*). Heredity. 2017;118:348–57. 27782121 10.1038/hdy.2016.105 PMC5345602

[145] Migalska M, Sebastian A, Radwan J. Profiling of the TCRβ repertoire in non-model species using high-throughput sequencing. Sci Rep. 2018;8:11613. 30072736 10.1038/s41598-018-30037-0 PMC6072738

[146] Migalska M, Sebastian A, Radwan J. Major histocompatibility complex class I diversity limits the repertoire of T cell receptors. Proc Natl Acad Sci. 2019;116:5021–6. 30796191 10.1073/pnas.1807864116 PMC6421458

[147] Mihalca AD, Sándor AD. The role of rodents in the ecology of Ixodes ricinus and associated pathogens in central and Eastern Europe. Front Cell Infect Microbiol. 2013;3:56. 24102049 10.3389/fcimb.2013.00056 PMC3787251

[148] Mills SC, Grapputo A, Jokinen I, Koskela E, Mappes T, Oksanen TA, Poikonen T. Testosterone-mediated effects on fitness-related phenotypic traits and fitness. Am Nat. 2009;173:475–87. 19236274 10.1086/597222

[149] Mills SC, Grapputo A, Jokinen I, Koskela E, Mappes T, Poikonen T. Fitness trade-offs mediated by immunosuppression costs in a small mammal. Evolution. 2010;64:166–79. 19686266 10.1111/j.1558-5646.2009.00820.x

[150] Mills SC, Koskela E, Mappes T. Intralocus sexual conflict for fitness: sexually antagonistic alleles for testosterone. Proc R Soc B Biol Sci. 2012;279:1889–95. 10.1098/rspb.2011.2340 PMC3311893 22171083

[151] Modlinska K, Pisula W. The Norway rat, from an obnoxious pest to a laboratory pet. Elife. 2020;9:50651. 10.7554/eLife.50651 PMC6968928 31948542

[152] Mokkonen M, Kokko H, Koskela E, Lehtonen J, Mappes T, Martiskainen H, Mills SC. Negative frequency-dependent selection of sexually antagonistic alleles in *Myodes glareolus*. Science. 2011;334:972–4. 22096197 10.1126/science.1208708

[153] Moulin TC, Covill LE, Itskov PM, Williams MJ, Schiöth HB. Rodent and fly models in behavioral neuroscience: an evaluation of methodological advances, comparative research, and future perspectives. Neurosci Biobehav Rev. 2021;120:1–12. 33242563 10.1016/j.neubiorev.2020.11.014

[154] National biodiversity data centre *Myodes glareolus*—species profile 2024 https://species.biodiversityireland.ie/profile.php?taxonId=119785&taxonDesignationGroupId=26 [accessed 21 April 2025].

[155] National research council (US) Committee for the update of the guide for the care and use of laboratory animals) guide for the care and use of laboratory animals, 8th edition. Washington (DC): National Academies Press (US); 2011.

[156] Nowak MA, Tarczy-Hornoch K, Austyn JM. The optimal number of major histocompatibility complex molecules in an individual. Proc Natl Acad Sci. 1992;89:10896–9. 1438295 10.1073/pnas.89.22.10896 PMC50449

[157] Nyholm NEI, Meurling P. Reproduction of the bank vole, *Clethrionomys glareolus*, in Northern and Southern Sweden during several seasons and in different phases of the vole population cycle. Holarct Ecol. 1979;2:12–20.

[158] Oksanen TA, Koivula M, Koskela E, Mappes T. The cost of reproduction induced by body size at birth and breeding density. Evolution. 2007;61:2822–31. 17924957 10.1111/j.1558-5646.2007.00245.x

[159] Oksanen TA, Koskela E, Mappes T. Hormonal manipulation of offspring number: maternal effort and reproductive costs. Evolution. 2002;56:1530–7. 12206251 10.1111/j.0014-3820.2002.tb01463.x

[160] Ołdakowski Ł, Wasiluk A, Sadowska ET, Koteja P, Taylor JRE. Reproduction is not costly in terms of oxidative stress. J Exp Biol. 2015;218(24):3901–10. 26519508 10.1242/jeb.126557

[161] Olsson GE, Leirs H, Henttonen H. Hantaviruses and their hosts in Europe: reservoirs here and there, but not everywhere? Vector Borne Zoonotic Dis. 2010;10:549–61. 20795916 10.1089/vbz.2009.0138

[162] Olsson GE, White N, Ahlm C, Elgh F, Verlemyr A-C, Juto P, Palo RT. Demographic factors associated with hantavirus infection in bank voles (*Clethrionomys glareolus*). Emerg Infect Dis. 2002;8:924–9. 12194768 10.3201/eid0809.020037 PMC2732544

[163] Orr HA. Fitness and its role in evolutionary genetics. Nat Rev Genet. 2009;10:531–9. 19546856 10.1038/nrg2603 PMC2753274

[164] Ostfeld R.S. 1991. The Ecology of Territoriality in Bank Voles a reply. Trends in Ecology and Evolution 6:301 10.1016/0169-5347(91)90011-L 21232488

[165] Paziewska A, Harris PD, Zwolińka L, Bajer A, Siński E. Differences in the ecology of Bartonella infections of *Apodemus flavicollis* and *Myodes glareolus* in a boreal forest. Parasitology. 2012;139:881–93. 22336264 10.1017/S0031182012000170

[166] Paziewska A, Zwolińska L, Harris PD, Bajer A, Siński E. Utilisation of rodent species by larvae and nymphs of hard ticks (Ixodidae) in two habitats in NE Poland. Exp Appl Acarol. 2010;50:79–91. 19421876 10.1007/s10493-009-9269-8

[167] Pekkarinen P, Heikkila J. Prey selection of the least weasel *Mustela nivalis* in the laboratory. Acta Theriol. 1997;42:179–88.

[168] Pemberton J, Bancroft D, Amos B. The ecology of territoriality in bank voles reply from R. Ostfeld. 1997;6:300–1.

[169] Perlman RL. Mouse models of human disease: an evolutionary perspective. Evol Med Public Health. 2016;2016(1):170–4. 27121451 10.1093/emph/eow014 PMC4875775

[170] Poikonen T, Koskela E, Mappes T, Mills SC. Infanticide in the evolution of reproductive synchrony: effects on reproductive success. Evolution. 2008;62:612–21. 17983462 10.1111/j.1558-5646.2007.00293.x

[171] Prévot-Julliard A.-C, Henttonen H, Yoccoz NG, Stenseth NC. Delayed maturation in female bank voles: optimal decision or social constraint? J Anim Ecol. 1999;68:684–97.

[172] Råberg L, Clough D, Hagström Å, Scherman K, Andersson M, Drews A, Strandh M, Tschirren B, Westerdahl H. MHC class II genotype-by-pathogen genotype interaction for infection prevalence in a natural rodent–borrelia system. Evolution. 2022;76:2067–75. 35909235 10.1111/evo.14590 PMC9541904

[173] Radwan J, Babik W, Kaufman J, Lenz TL, Winternitz J. Advances in the evolutionary understanding of MHC polymorphism. Trends Genet. 2020;36:298–311. 32044115 10.1016/j.tig.2020.01.008

[174] Redeker S, Andersen LW, Pertoldi C, Madsen AB, Jensen TS, Jorgensen JM. Genetic structure, habitat fragmentation and bottlenecks in Danish bank votes (*Clethrionomys glareolus*). Mamm Biol. 2006;71:144–58.

[175] Reijniers J, Tersago K, Borremans B, Hartemink N, Voutilainen L, Henttonen H, Leirs H. Why hantavirus prevalence does not always increase with host density: modeling the role of host spatial behavior and maternal antibodies. Front Cell Infect Microbiol. 2020;10:536660. 33134187 10.3389/fcimb.2020.536660 PMC7550670

[176] Reil D, Imholt C, Eccard JA, Jacob J. Beech fructification and bank vole population dynamics—combined analyses of promoters of human puumala virus infections in Germany. PLoS ONE. 2015;10: e0134124. 26214509 10.1371/journal.pone.0134124 PMC4516252

[177] Röhrs S, Begeman L, Straub BK, Boadella M, Hanke D, Wernike K, Drewes S, Hoffmann B, Keller M, Drexler JF, Drosten C, Höper D, Kuiken T, Ulrich RG, Beer M. The bank vole (*Clethrionomys glareolus*)—small animal model for hepacivirus infection. Viruses. 2021;13:2421. 34960690 10.3390/v13122421 PMC8708279

[178] Roy HE, Tricarico E, Hassall R, Johns CA, Roy KA, Scalera R, Smith KG, Purse BV. The role of invasive alien species in the emergence and spread of zoonoses. Biol Invasions. 2023;25:1249–64. 36570096 10.1007/s10530-022-02978-1 PMC9763809

[179] Różańska-Wróbel J, Migalska M, Urbanowicz A, Grzybek M, Rego ROM, Bajer A, Dwuznik-Szarek D, Alsarraf M, Behnke-Borowczyk J, Behnke JM, Radwan J. Interplay between vertebrate adaptive immunity and bacterial infectivity genes: bank vole MHC versus *Borrelia afzelii* OspC. Mol Ecol. 2024;33(21):e17534. 39314079 10.1111/mec.17534

[180] Rudolf AM, Dańko MJ, Sadowska ET, Dheyongera G, Koteja P. Age-related changes of physiological performance and survivorship of bank voles selected for high aerobic capacity. Exp Gerontol. 2017;98:70–9. 28803134 10.1016/j.exger.2017.08.007

[181] Sadowska E, Labocha M, Baliga K, Stanisz A, Wróblewska A, Jagusiak W, Koteja P. Genetic correlations between basal and maximum metabolic rates in a wild rodent: consequences for evolution of endothermy. Evolution. 2005;59:672–81. 15856708

[182] Sadowska ET, Baliga-Klimczyk K, Chrząścik KM, Koteja P. Laboratory model of adaptive radiation: a selection experiment in the bank vole. Physiol Biochem Zool. 2008;81:627–40. 18781839 10.1086/590164

[183] Sadowska ET, Baliga-Klimczyk K, Labocha MK, Koteja P. Genetic correlations in a wild rodent: grass-eaters and fast-growers evolve high basal metabolic rates. Evolution. 2009;63:1530–9. 19187250 10.1111/j.1558-5646.2009.00641.x

[184] Sadowska ET, Król E, Chrzascik KM, Rudolf AM, Speakman JR, Koteja P. Limits to sustained energy intake. XXIII. Does heat dissipation capacity limit the energy budget of lactating bank voles ? J Exp Biol. 2016;219(6):805–15. 26747907 10.1242/jeb.134437

[185] Sadowska ET, Stawski C, Rudolf A, Dheyongera G, Chrząścik KM, Baliga-Klimczyk K, Koteja P. Evolution of basal metabolic rate in bank voles from a multidirectional selection experiment. Proc R Soc B Biol Sci. 2015;282:20150025. 10.1098/rspb.2015.0025 PMC4426621 25876844

[186] Salvador AR, Guivier E, Xuéreb A, Chaval Y, Cadet P, Poulle M-L, Sironen T, Voutilainen L, Henttonen H, Cosson J-F, Charbonnel N. Concomitant influence of helminth infection and landscape on the distribution of *Puumala hantavirus* in its reservoir *Myodes glareolus*. BMC Microbiol. 2011;11:30. 21303497 10.1186/1471-2180-11-30 PMC3040693

[187] Schønecker B, Freimanis T, Sørensen IV. Diabetes in Danish bank voles (*M. glareolus*): survivorship, influence on weight, and evaluation of polydipsia as a screening tool for hyperglycaemia. PLoS ONE. 2011;6:22893. 10.1371/journal.pone.0022893 PMC3150384 21829666

[188] Schroderus E, Koivula M, Koskela E, Mappes T, Oksanen TA, Poikonen T. Can number and size of offspring increase simultaneously? A central life-history trade-off reconsidered. BMC Evol Biol. 2012;12:44. 22462658 10.1186/1471-2148-12-44 PMC3353187

[189] Searle JB, Kotlík P, Rambau RV, Marková S, Herman JS, McDevitt AD. The celtic fringe of Britain: insights from small mammal phylogeography. Proc R Soc B Biol Sci. 2009;276:4287–94. 10.1098/rspb.2009.1422 PMC2817114 19793757

[190] Shenbrot GI, Krasnov BR. An atlas of the geographic distribution of the arvicoline rodents of the world (Rodentia, Muridae: Arvicolinae). Sofia: Pensoft Publishers; 2005.

[191] Shore R, Hare EJ. Bank Vole. In: Harris S, Yalden DW, editors. Mammals of the British Isles: handbook. 4th ed. The Mammal Society; 2008. p. 88–99.

[192] Siński E, Pawełczyk A, Bajer A, Behnke JM. Abundance of wild rodents, ticks and environmental risk of Lyme borreliosis: a longitudinal study in an area of Mazury Lakes district of Poland. Ann Agric Environ Med. 2006;13:295–300. 17196004

[193] Smal CM. The diet of the Barn Owl Tyto alba in southern Ireland, with reference to a recently introduced prey species—the bank vole *Clethrionomys glareolus*. Bird Study. 1987;34:113–25.

[194] Sommer S. The importance of immune gene variability (MHC) in evolutionary ecology and conservation. Front Zool. 2005;2:16. 16242022 10.1186/1742-9994-2-16 PMC1282567

[195] Spitzenberger F. The atlas of European mammals. London: Academic Press; 1999.

[196] Standley A, Xie J, Lau AW, Grote L, Gifford AJ. Working with miraculous mice: mus musculus as a model organism. Curr Protoc. 2024;4(10):e70021. 39435766 10.1002/cpz1.70021

[197] Stawski C, Koteja P, Sadowska ET. A shift in the thermoregulatory curve as a result of selection for high activity-related aerobic metabolism. Front Physiol. 2017;8:1070. 29326604 10.3389/fphys.2017.01070 PMC5741638

[198] Stawski C, Koteja P, Sadowska ET, Jefimow M, Wojciechowski MS. Selection for high activity-related aerobic metabolism does not alter the capacity of non-shivering thermogenesis in bank voles. Comp Biochem Physiol A Mol Integr Physiol. 2015;180:51–6. 25446149 10.1016/j.cbpa.2014.11.003

[199] Stopka P, Macdonald DW. Way-marking behaviour: an aid to spatial navigation in the wood mouse (*Apodemus sylvaticus*). BMC Ecol. 2003;3:3. 12697070 10.1186/1472-6785-3-3 PMC154096

[200] Stopková R, Zdráhal Z, Ryba Š, Šedo O, Šandera M, Stopka P. Novel OBP genes similar to hamster Aphrodisin in the bank vole *Myodes glareolus*. BMC Genomics. 2010;11:45. 20085627 10.1186/1471-2164-11-45 PMC2824723

[201] Storz JF, Bridgham JT, Kelly SA, Garland T. Genetic approaches in comparative and evolutionary physiology. Am J Physiol Regul Integr Comp Physiol. 2015;309:R197–214. 26041111 10.1152/ajpregu.00100.2015 PMC4525326

[202] Storz JF, Hoffmann FG, Opazo JC, Moriyama H. Adaptive functional divergence among triplicated α-Globin genes in rodents. Genetics. 2008;178:1623–38. 18245844 10.1534/genetics.107.080903 PMC2278084

[203] Strážnická M, Marková S, Searle J, Kotlík P. Playing hide-and-seek in beta-globin genes: gene conversion transferring a beneficial mutation between differentially expressed gene duplicates. Genes. 2018;9:492. 30321987 10.3390/genes9100492 PMC6209878

[204] Stuart P, Mirimin L, Cross TF, Sleeman DP, Buckley JN, Telfer S, Birtles RJ, Kotlik P, Searle JB. The origin of Irish bank voles *Clethrionomys glareolus* assessed by mitochondrial DNA analysis. Ir Nat J. 2007;28:440–6.

[205] Stuart P, Paredis L, Henttonen H, Lawton C, Ochoa Torres CA, Holland CV. The hidden faces of a biological invasion: parasite dynamics of invaders and natives. Int J Parasitol. 2020;50:111–23. 31981672 10.1016/j.ijpara.2019.11.003

[206] Tarnowska E, Niedziałkowska M, Stojak J, Jędrzejewska B. Polymorphism of TLR2 in bank vole populations in North Eastern Poland is not associated with *Borrelia afzelii* infection prevalence. Mammal Res. 2020;65:779–91.

[207] Tegelström H. Transfer of mitochondrial DNA from the northern red-backed vole (*Clethrionomys rutilus*) to the bank vole (*C. glareolus*). J Mol Evol. 1987;24:218–27. 3106637 10.1007/BF02111235

[208] Telfer S, Bennett M, Bown K, Cavanagh R, Crespin L, Hazel S, Jones T, Begon M. The effects of cowpox virus on survival in natural rodent populations: increases and decreases. J Anim Ecol. 2002;71:558–568. 10.1046/j.1365-2656.2002.00623.x.

[209] Tersago K, Verhagen R, Servais A, Heyman P, Ducoffre G, Leirs H. Hantavirus disease (nephropathia epidemica) in Belgium: effects of tree seed production and climate. Epidemiol Infect. 2009;137:250–6. 18606026 10.1017/S0950268808000940

[210] Tonteri E, Jääskeläinen AE, Tikkakoski T, Voutilainen L, Niemimaa J, Henttonen H, Vaheri A, Vapalahti O. Tick-borne encephalitis virus in wild rodents in winter, Finland, 2008–2009. Emerg Infect Dis. 2011;17:72–5. 21192857 10.3201/eid1701.100051 PMC3204619

[211] Tschirren B, Andersson M, Scherman K, Westerdahl H, Mittl PRE, Råberg L. Polymorphisms at the innate immune receptor TLR2 are associated with Borrelia infection in a wild rodent population. Proc R Soc B Biol Sci. 2013;280:20130364. 10.1098/rspb.2013.0364 PMC3619520 23554395

[212] Tuomi J, Agrell J, Mappes T. On the evolutionary stability of female infanticide. Behav Ecol Sociobiol. 1997;40:227–33.

[213] Ulrich L, Michelitsch A, Halwe N, Wernike K, Hoffmann D, Beer M. Experimental SARS-CoV-2 Infection of Bank Voles. Emerg Infect Dis. 2021;27:1193–5. 33754987 10.3201/eid2704.204945 PMC8007283

[214] Upham N, Burgin C, Widness J, Liphardt S, Parker C, Becker M, Rochon I, Huckaby D. Mammal diversity database. American Society of Mammalogists; 2023.

[215] Vaheri A, Henttonen H, Voutilainen L, Mustonen J, Sironen T, Vapalahti O. Hantavirus infections in Europe and their impact on public health. Rev Med Virol. 2013;23:35–49. 22761056 10.1002/rmv.1722

[216] Välimaa H, Niemimaa J, Oksanen A, Henttonen H. *Sylvatic trichinella* reservoir not found among voles in Finland. Acta Vet Scand. 2010;52:S15.

[217] Vapalahti O, Mustonen J, Lundkvist A, Henttonen H, Plyusnin A, Vaheri A. Hantavirus infections in Europe. Lancet Infect Dis. 2003;3:653–61. 14522264 10.1016/s1473-3099(03)00774-6

[218] Villano JS, Follo JM, Chappell MG, Collins MT. Personal protective equipment in animal research. Comp Med. 2017;67:203–14. 28662749 PMC5482512

[219] Viro P, Sulkava S. Food of the bank vole in Northern Finish Spruce forest. Acta Theriol. 1985;30:259–66.

[220] Voutilainen L, Kallio ER, Niemimaa J, Vapalahti O, Henttonen H. Temporal dynamics of *Puumala hantavirus* infection in cyclic populations of bank voles. Sci Rep. 2016;6:21323. 26887639 10.1038/srep21323 PMC4758042

[221] Voutilainen L, Savola S, Kallio ER, Laakkonen J, Vaheri A, Vapalahti O, Henttonen H. Environmental change and disease dynamics: effects of intensive forest management on puumala hantavirus infection in boreal bank vole populations. PLoS ONE. 2012;7: e39452. 22745755 10.1371/journal.pone.0039452 PMC3380007

[222] Voutilainen L, Sironen T, Tonteri E, Bäck AT, Razzauti M, Karlsson M, Wahlström M, Niemimaa J, Henttonen H, Lundkvist Å. Life-long shedding of *Puumala hantavirus* in wild bank voles (*Myodes glareolus*). J Gen Virol. 2015;96:1238–47. 25701819 10.1099/vir.0.000076

[223] Vucetich JA, Nelson MP, Bruskotter JT. What drives declining support for long-term ecological research? Bioscience. 2020;70:168–73.

[224] Wasberg A, Raghwani J, Li J, Pettersson JH-O, Lindahl JF, Lundkvist Å, Ling J. Discovery of a novel coronavirus in swedish bank voles (*Myodes glareolus*). Viruses. 2022;14:1205. 35746677 10.3390/v14061205 PMC9230040

[225] Watts CHS. The foods eaten by wood mice (*Apodemus sylvaticus*) and bank voles (*Clethrionomys glareolus*) in wytham woods, Berkshire. The J Anim Ecol. 1968;37:25.

[226] Welc-Faleciak R, Bajer A, Behnke JM, Siński E. Effects of host diversity and the community composition of hard ticks (Ixodidae) on *Babesia microti* infection. Int J Med Microbiol. 2008;298:235–42.

[227] Welc-Falęciak R, Bajer A, Behnke JM, Siński E. The ecology of Bartonella spp. infections in two rodent communities in the Mazury Lake District region of Poland. Parasitology. 2010;137:1069–77. 20388232 10.1017/S0031182009992058

[228] White TA, Lundy MG, Montgomery WI, Montgomery S, Perkins SE, Lawton C, Meehan JM, Hayden TJ, Heckel G, Reid N, Searle JB. Range expansion in an invasive small mammal: influence of life-history and habitat quality. Biol Invasions. 2012;14:2203–15.

[229] Ylonen H, Viitala J. Social overwintering and food distribution in the bank vole *Clethrionomys glareolus*. Ecography. 1991;14:131–7.

[230] Ylönen H, Viitala J, Mappes T. How much do avian predators influence cyclic bank vole populations? An experiment during a peak year. Ann Zool Fenn. 1991;28:1–6.

[231] Yoccoz NG, Stenseth NChR, Henttonen H, Prévot-Julliard A. Effects of food addition on the seasonal density-dependent structure of bank vole *Clethrionomys glareolus* populations. J Anim Ecol. 2001;70:713–20.

[232] Young HS, Parker IM, Gilbert GS, Sofia Guerra A, Nunn CL. Introduced species, disease ecology, and biodiversity-disease relationships. Trends Ecol Evol. 2017;32:41–54. 28029377 10.1016/j.tree.2016.09.008

